# Microbiota and Lifestyle: A Special Focus on Diet

**DOI:** 10.3390/nu12061776

**Published:** 2020-06-15

**Authors:** Noemí Redondo-Useros, Esther Nova, Natalia González-Zancada, Ligia E. Díaz, Sonia Gómez-Martínez, Ascensión Marcos

**Affiliations:** Immunonutrition Group, Department of Metabolism and Nutrition, Institute of Food Science, Technology and Nutrition (ICTAN), Spanish National Research Council (CSIC), Jose Antonio Novais, St.10, 28040 Madrid, Spain; noemi_redondo@ictan.csic.es (N.R.-U.); enova@ictan.csic.es (E.N.); n.gonzalez@ictan.csic.es (N.G.-Z.); ldiaz@ictan.csic.es (L.E.D.); sgomez@ictan.csic.es (S.G.-M.)

**Keywords:** gut microbiota, healthy adults, diet, lifestyle, inter-individual and intra-individual factors

## Abstract

It is widely known that a good balance and healthy function for bacteria groups in the colon are necessary to maintain homeostasis and preserve health. However, the lack of consensus on what defines a healthy gut microbiota and the multitude of factors that influence human gut microbiota composition complicate the development of appropriate dietary recommendations for our gut microbiota. Furthermore, the varied response to the intake of probiotics and prebiotics observed in healthy adults suggests the existence of potential inter- and intra-individual factors, which might account for gut microbiota changes to a greater extent than diet. The changing dietary habits worldwide involving consumption of processed foods containing artificial ingredients, such as sweeteners; the coincident rise in emotional disorders; and the worsening of other lifestyle habits, such as smoking habits, drug consumption, and sleep, can together contribute to gut dysbiosis and health impairment, as well as the development of chronic diseases. This review summarizes the current literature on the effects of specific dietary ingredients (probiotics, prebiotics, alcohol, refined sugars and sweeteners, fats) in the gut microbiota of healthy adults and the potential inter- and intra-individual factors involved, as well as the influence of other potential lifestyle factors that are dramatically increasing nowadays.

## 1. Introduction

**Figure 1:**
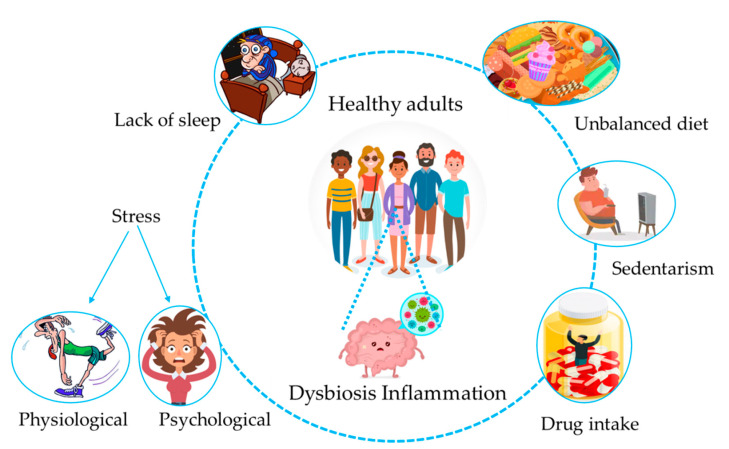
Interplay between lifestyle habits and the gut microbiota. A poor lifestyle characterized by an unbalanced diet, sedentarism, chronic intake of drugs, a lack of sleep, and physiological or psychological stress can lead to asymptomatic dysbiosis, and thus to inflammatory states, all contributing to disease development in the long term.

Nowadays, the high incidence of non-communicable diseases (NCDs) such as obesity, inflammatory bowel diseases, and allergies has become an important health issue due to its fast growth in developed countries [1]. Pregnancy and the first 1000 days of life are considered to be the most essential and critical periods in terms of developing NCDs [2] due to the high plasticity of the metabolic, immunological, and cognitive functions towards environmental factors. Indeed, the initial microbial contact and nutritional status also have special importance in this period [3]. The notion that lifestyle plays an important role in NCD development came from the formulation in 1989 of the “hygiene hypothesis”, which related the increased incidence of immune-related diseases with an use of antibiotics and consumption of sterilized foods [4]. Specifically, an extremely clean environment during early childhood leads to a lack of exposure to germs and infections, reducing microbial diversity and leading to a lower number of “old friends”, or essential bacteria for an optimal immune response, such as helminths and pseudo-commensal bacteria present in water and foods, which are necessary for optimal Th2 and regulatory responses. Therefore, the uncorrected activation of the immune system towards a more Th1-inflammatory response can induce gut dysbiosis, and thus the development of inflammatory chronic diseases [5]. Both inflammation and gut dysbiosis can also be triggered by genetics, unbalanced diets, and stressful conditions [6]. A healthy gut microbiota is characterized by resistance and resilience, defined as the ability to resist an external perturbation and to return to the pre-perturbation state after a change occurs, respectively [7]; therefore, gut microbiota exhibiting high plasticity towards the environment [8] could be a double-edged sword (Figure 1). Indeed, if an external factor is stronger than the stability of the gut ecosystem to an extent exceeding the resistance and resilience capabilities [9], the return of the microbial community to the previous state can be compromised, leading to the development of permanent dysbiotic states [5,10] in either the bacteria composition or functionality [9,11]. At this point, whereby dysbiosis predominates over balanced states, the consequences can be detrimental for host health [9].

The most recent literature reporting a one-to-one ratio for the contribution of bacteria/human cells into the organism shows the huge preponderant role of the gut microbiota in the human body. Therefore, dysbiosis is a very important condition that requires consideration when studying any health disorder [12]. Indeed, the close link between gut bacteria and epithelial and immune cells means that any damage to either the composition or functionality of gut communities will negatively affect cell functionality. For instance, there are several fundamental roles exerted by the gut microbiota that might be impaired under dysbiotic states, including the synthesis of metabolites (short-chain fatty acids (SCFA), vitamins, or bile acids), immunomodulation, pathogen defense, and brain functionality [13,14]. Impairment of the above roles can lead to the homeostatic processes of the human body being damaged [15], promoting the development of chronic oxidative and inflammatory processes over time, and thus disease stabilization [16]. One good example is the practice of long-term dietary habits characterized by the high intake of refined sugars or fats and their relationship with a proinflammatory intestinal milieu and the depletion of beneficial bacteria, together with the enrichment in pathogenic and proinflammatory microbes [17]. These changes can impair butyrate production and induce inflammatory responses, damaging the intestinal barrier permeability and promoting uncomfortable intestinal symptoms, such as flatulence or bloating [18].

In this scenario, apparently healthy adults without diagnosed inflammatory chronic diseases [19] can be considered key targets to evaluate the response of a “healthy” gut microbiota against lifestyle factors. Pioneering metagenomic studies performed in healthy adults, such as the Human Microbiome Project (HMP), defined a healthy gut microbiota as “a core set of microbial taxa universally present in healthy individuals who lack overt disease phenotypes under the hypothesis that the absence of such microbes would indicate dysbiosis” [13]. The HMP also revealed that each individual harbors 600,000 microbial genes in the gastrointestinal (GI) tract, half of which are shared among individuals. Ninety-nine percent of these have a bacterial origin, with the rest being from Archaea and a very small proportion being of viral origin. The core bacterial microbial genes mainly belong to the Firmicutes and Bacteroidetes phyla, followed by Actinobacteria, Proteobacteria, Fusobacteria, and Verrucomicrobia to lesser extents [20]. In addition, each individual harbors a particular and variable number of bacterial species that are rare among individuals, defined as the “variable” gut microbiota [21], determining the high variability found in the gut microbiota composition among healthy individuals [22]. The variable gut microbiota includes autochthonous species that colonize the intestine, while others are allochthonous species that have shown the ability to transiently integrate into the resident gut microbiota and are mainly derived from the diet [23]. The yogurt starters *Lactobacillus delbrueckii* ssp. *bulgaricus* and *Streptococcus thermophilus*, as well as other lactic acid bacteria and Bifidobacteria in probiotic fermented milks, such as *Lactobacillus casei* and *Bifodobacterium animalis,* are some examples of transient microorganisms [24,25,26]. The high variability found among healthy individuals in the HMP and others [13,20,27] called into question the idea of a healthy core microbiome composed by specific microorganisms. In this respect, the observation that microbial functions performed by different microorganisms are well-conserved among different healthy individuals has led to a new definition of a healthy gut microbiota as a “functional core”, or a set of microbial genes performing metabolic functions that are preserved among healthy individuals, rather than common microorganisms [24]. Qin et al., as part of the HMP project, revealed two types of bacterial functions: (1) Those functions necessary for a bacteria to thrive in a gut context (the minimal gut genome), such as metabolic pathways (central carbon metabolism and amino acid synthesis). (2) Those functions involved in the homeostasis of the whole ecosystem and encoded across many species (the minimal gut metagenome), such as the adhesion to host proteins or biodegradation of complex sugars and glycans harvested from the host diet or intestinal lining [20]. Therefore, both composition and functionality are useful biomarkers of gut health. Apparently, healthy adults can experience health disturbances characterized by proinflammatory states and gut microbiota alterations, including slight constipation, overweight, or metabolic disturbances, such as high plasma cholesterol levels [19], suggesting that these conditions can be early stages of the beginning and progression of inflammatory chronic diseases [28]. Therefore, the study of diet–gut microbiota interactions in healthy adults could provide new insights about the mechanistic process by which the “healthy” gut microbiota responds to environmental factors and to ascertain if a particular change in the gut microbiota can be considered the trigger of any chronic immune-related disorder. For this purpose, the main goal of this work is to perform a systemic review of diet effects in the gut microbiota composition of healthy adults, particularly from probiotics, yogurts, prebiotics (fiber and polyphenols), alcoholic beverages, sweeteners, and fats. The effects of other lifestyle factors, such as physiological (extreme physical activity and sleep alterations) and psychological (emotional disturbances) stress, physical activity, drug intake, air quality, and pollutants and smoking habits, have been also reviewed, as well as the relevant factors involved in the diet–gut microbiota interactions.

## 2. Diet

Diet has been suggested as one of the main geographical factors responsible for gut microbiota differences across the world [29]. In particular, long-term dietary habits, which include not only the specific composition of nutrients, but also meal times and food behaviors, account for deeper and chronic changes in the gut microbiota than short dietary interventions [30,31,32,33]. The main dietary patterns worldwide are the Western, Mediterranean, and Vegetarian diets, which differ widely in their composition of foods and nutrients. Indeed, the Western lifestyle typical of occidental countries is characterized by high amounts of processed foods rich in refined fats and sugars, salt, and animal proteins [34], whereas the Mediterranean diet, common of populations living around the Mediterranean Sea, is mainly composed of fruits, vegetables, legumes, nuts, and olive oil, along with moderate consumption of fish and dairy products. Finally, vegetarian diets exclude the consumption of animal products such as fish, meat, and seafood, and mainly consist of fiber-enriched foods and vegetal proteins, together with healthy fats. Existing evidence has pointed out the different consequences on health derived from each dietary pattern [34,35,36], which raises the necessity of a deeper study of their particular nutrients or ingredients in order to ascertain the specific effects on the gut microbiota composition.

### 2.1. Methodology

We performed a systematic review of diet effects on the gut microbiota of healthy adults, according to the Preferred Reporting Items for Systematic Reviews and Meta-Analyses (PRISMA) statement guidelines.

#### 2.1.1. Search Strategy

The literature search was performed in Pubmed and SCOPUS databases from January 2019 to June 2019, with the following strategy: “Probiotic” AND “healthy adults” or “human” AND “microbiota” or “ feces”; “Yogurt” AND “healthy adults” AND “microbiota”; “Prebiotic” or “Fiber” AND “healthy adults” or “adults” AND “gut microbiota” or “microbiota”; “Polyphenols” or “Flavonoids” AND “adults” AND “microbiota” or “feces”; “Red wine” or “Beer” or “Alcohol” AND “human” AND “gut microbiota” or “microbiota”; “artificial sweeteners” AND “human” AND “feces”; “Fats” or “Dairy” AND “healthy adults” AND “gut microbiota”. Studies in English language and published beyond 2006 were included in the systematic review.

#### 2.1.2. Selection Criteria

We made a first exclusion procedure with the following criteria: in vitro, animal or human studies involving infants or elderly people were excluded, as well as disease conditions (obesity, diabetes, cardiovascular, inflammatory bowel diseases, celiac disease, cancer, and allergies). Then, we revised the selected studies and performed a second round of exclusion: small sample size (*n* < 10 subjects), short duration of treatment (<1 week), microbiota composition was not the main studied variable, and incomplete demographic data (age, gender and body mass index (BMI) not reported). In addition, for both probiotic and prebiotic studies, we excluded observational trials and studies performed with symbiotics.

**Figure 2:**
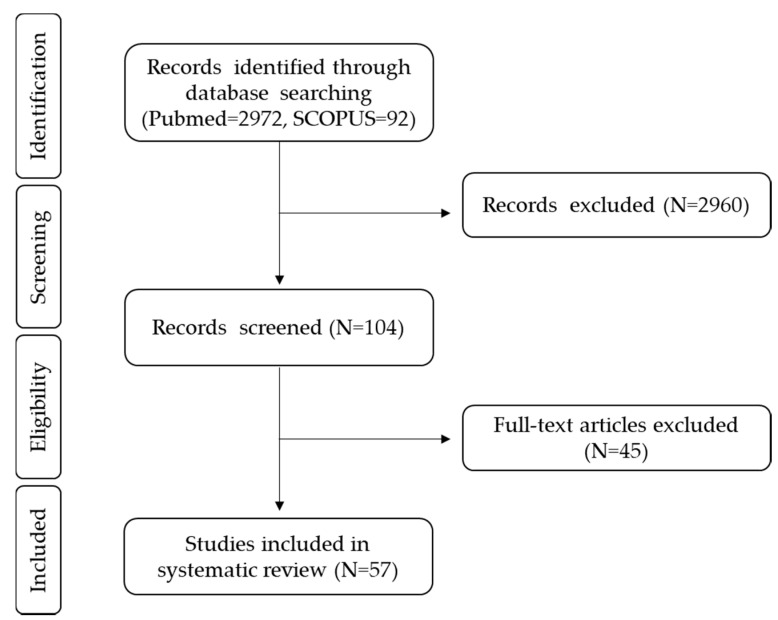
PRISMA flow chart. PRISMA flow chart of studies focused on diet effects on the gut microbiota of healthy adults.

Finally, we included the randomized clinical trials following the population, intervention, comparison, outcome (PICO) criteria: healthy adults as the study population; probiotics, yogurts, fiber, polyphenols, alcoholic beverages, sweeteners, and fats as the intervention; placebo and basal points before the dietary intervention as comparators, and gut microbiota changes after dietary treatment as the main outcome, including changes in the levels of bacteria groups and also in α and β-diversity measures. In order to provide more information in novel fields such as sweeteners and alcohol, observational studies using the PICO criteria were also included. The complete process is explained in Figure 2, according to the PRISMA flow chart.

#### 2.1.3. Data Collection Process

Data extracted from each trial are presented in Table 1, Table 2, Table 3, Table 4 and Table 5, according to the following process: (1) Treatment: Dose, duration, and type of placebo for all studies, specifying the type of probiotic strain, fiber, and polyphenols when necessary. (2) Study type: randomization process, single- or double-blinded, observational or intervention, crossover or parallel. (3) Study subjects: sample size, gender, age, and BMI. (4) Microbiota analysis technique: cellular cultures, quantitative PCR (qPCR), fluorescence in situ hybridization (FISH), and 16s RNA sequencing methods (16s RNA gene regions, sequencing platform). (5) Results were organised according to three aspects: differences after the dietary treatment in comparison to basal values, differences between treatment groups if two or more groups were included in the study design, and differences after the dietary treatment compared to the control group.

### 2.2. Results and Discussion

#### 2.2.1. Probiotics

Probiotics are defined as “live microorganisms, which when administered in adequate amounts, confer a health benefit on the host”. *Bifidobacterium* and *Lactobacillus* species are historically the most used, but in recent years, species from *Lactococcus*, *Streptococcus*, *Pediococcus*, and some yeast, such as *Saccharomyces boulardii*, have gained special attention [92]. Probiotics are usually consumed as capsules, but fermented milks are also a favorable vehicle for the strains, resulting in a product with good nutritional value and palatability [93]. Most probiotic strains transiently colonize the gut and disappear in feces in a few days after intake cessation [26,94], thus effects of probiotics on the gut microbiota depend on the continuous intake of the strains. After ingestion, probiotic strains must survive the GI conditions and reach the colon to exert their immunomodulatory effects. Indeed, one of the most evidenced effects of probiotics is their ability to prevent GI and respiratory infections, which are mainly driven through the inhibition of pathogen growth in the gut, the promotion of the intestinal barrier integrity, and the stimulation of the innate and specific immune system. The last mechanism can be achieved either by direct mechanisms or by interaction with the lumen commensal bacteria. In the first case, there may be an interaction with epithelial cells or immune cells of the lamina propia, which possess specialized receptors, such as toll-like receptors (TLRs), capable of detecting specific components of the bacteria cell wall (lipopolysaccharides (LPS) or peptidoglycans) [95]. In the second case, bacteria can modulate the expression of pathogen-associated molecular patterns (PAMPs), and consequently have a direct effect on the activity of immune cells. Indirect mechanisms, such as the production of microbial metabolites such as SCFA by probiotic strains, can also modulate the immune system and the composition of bacteria groups in the gut [96].

The revised interventional studies with probiotics were divided in two groups: studies showing changes in those bacteria taxonomically related to the probiotic strains consumed, and studies showing changes in the overall microbial composition, such as α or β-diversity, or in bacteria groups differing from ingested probiotic strains.

Regarding changes in the levels of bacteria taxonomically related to the ingested probiotic strains, the most common finding in the reviewed studies is the increase in the fecal *Lactobacillus* [39,45,47,48,49,50,54] or *Bifidobacterium* levels [41,49,50], particularly after the intake of *Lactobacillus* strains, suggesting the existence of bacterial cross-feeding mechanisms in the intestinal niche. In this respect, there is evidence that some *Lactobacillus* strains can either degrade dietary carbohydrates or host mucin polysaccharides. For instance, *L. paracasei* 8700 can breakdown inulin into short fractions of carbohydrates, which in turn serve as substrates for *B. longum* LMG 11,047 [97,98]. On the contrary, no changes in *Lactobacillus* or *Bifidobacterium* levels have been found in other studies [40,42,51,52]. The different methodology used to analyze the gut microbiota can influence the interpretation of the results, as observed in the overestimation or underestimation of bacteria levels in traditional bacterial cultures [40,50].

Bacterial diversity is a common measure used to quantify the number (richness) and abundance (evenness) of species that are present in an ecosystem, and is a useful biomarker of the overall gut microbiota composition. The α-diversity indexes, such as Shannon or Simpson indexes, are measures of how evenly the microbes are distributed in a sample, whereas β-diversity shows the differences in taxonomic abundance profiles between different ecosystems or sample groups, calculated by Unifrac, Jaccard, and Bray–Curtis distances, among others [43]. Whereas a high bacterial diversity has been associated to a better metabolic profile and a good health status, a loss in bacterial diversity is a typical feature of certain metabolic disorders, such as obesity [99]. According to a recent review of randomized clinical trials in healthy adults, there is a lack of evidence to conclude whether or not there is an effect of probiotics on the gut microbiota composition of healthy adults. Small sample sizes, the use of different probiotic strains, and the use of low-resolution methods to analyze the gut microbiota are some of the main factors that make it difficult to reach clear conclusions [100]. Some of the reviewed studies showed no changes in either β-diversity or the levels of the bacteria groups analyzed [26], but others showed changes in specific bacteria groups despite bacterial diversity remaining unaltered. For instance, Kim et al. revealed higher levels of *Streptococcus salivarius*, *Eubacterium rectale* and *Fecalibacterium prausnitzii* after the intake of *Lactobacillus* and *Bifidobacterium* probiotic strains, whereas Bacteroidetes species were more affected by *Bifidobacterium* strains [101]. In addition, Burton et al. reported lower levels of *B. kashiwanohense* and *B. pseudocatenulatum* but higher levels of *Intestinibacter bartletti* after the intake of *L. rhamnosus* GG over 2 weeks compared to placebo [52]. However, different effects have been observed after the intake of other strains of *L. rhamnosus* (HN001), which induced changes at the species level and decreased Firmicutes and Proteobacteria levels [44]. The intake of *L. kefir* LKF01 over one month decreased Firmicutes, Bacteroides, and Proteobacteria phyla, as well as several bacteria groups [45], while *B. bifidum* Bb ingested over 4 weeks decreased *Prevotellaceae, Rikenellaceae*, and *Ruminococaceae* levels [46]. On the contrary, other studies have shown changes in both β-diversity and the levels of principal bacteria groups [41,42,53]. Volokh et al. pointed out that the intake of *B. animalis* ssp. *lactis* over one month did not change α-diversity but increased β-diversity and the levels of *Bifidobacterium*, *Streptococcus*, *Catenibacterium*, *Slackia, Collinsella*, and *Adlercreutzia*, whereas *Lachnoclostridium*, *Roseburia*, and *Acidaminococcaceae* levels decreased [53]. The intake of *L. paracasei* DG also increased β-diversity, as well as *Coprococcus* levels, but decreased Proteobacteria and *B. coccoides* levels [42]. In addition, the intake of *L. Zhang* over 4 weeks increased β-diversity, *Prevotella*, and *Fecalibacterium* levels, while *Enterobacter*, *Blautia-coccoides*, and *Phascolarctobacterium* levels decreased [41] (Table 1). In conclusion, the high variability found in the response of the gut microbiota to the intake of different probiotics reflects the well-known assumption that probiotic effects are strain-specific, and the existence of intra-subject and methodological factors has an important role in this respect, as exposed below in Section 5.

#### 2.2.2. Yogurt

Yogurt is defined as “the product obtained from the fermentation of milk by the starters *Lactobacillus delbrueccki* ssp. *bulgaricus* and *Streptococcus thermophilus*, which needs to be viable in a minimal dose of 10^7^ cfu/g” (CODEX STAN 243–2003). Yogurt intake has been related to a better diet quality, partially attributed to its high nutritional value [102]. Indeed, yogurt is a complex food matrix composed of high amounts of calcium, phosphorous, vitamins B, bioactive peptides, essential fatty acids (FAs), and lactic acid bacteria (LAB). Of all these ingredients, bacterial and FA content seem to be responsible for the attributed benefits of yogurt on the immune and gut health [103,104,105]. Yogurt cultures have been related to an improvement of intestinal disturbances, such as constipation or diarrhea [103]. The mechanisms seem to be similar to those mentioned above related to probiotic strains and to the creation of a beneficial gut environment. This outcome could be due to the interactions between yogurt cultures and the beneficial bacteria, or indirect mechanisms through the production of certain metabolites, such as bioactive peptides, which promote mucin synthesis and the enhancement of the intestinal barrier, leading to better protection against pathogens [106]. One of the basic premises of their effects on health is that live yogurt cultures need to survive the GI conditions and reach the colon in order to interact with the gut microbiota or immune cells. However, the literature shows controversy in the detection of yogurt strains in stool samples, with some positive results [107,108], but others results failing to detect necessary levels of the strains in the feces [38,109], suggesting that other factors such as the methodology, the type of strain, and the dose employed could influence the results [110]. Research on yogurt effects in the gut microbiota of healthy adults is very scarce, and the scientific literature is still very controversial, as exposed in Table 1. Uyeno et al. revealed higher levels of the *C. coccoides-E. rectale* group and lower levels of *Bacteroides-Prevotella* after the intake of fresh yogurt [37]. García-Albiach et al. showed that the intake of both fresh and pasteurized yogurt decreased *Bacteroides* levels, suggesting that other yogurt ingredients impacted these findings. In addition, the levels of *Lactobacillus* were higher after the intake of fresh yogurt compared to pasteurized yogurt, possibly due to yogurt starters [38]. The current evidence on the effects of fats on the gut microbiota suggests that the fatty acid content of yogurt could possibly play a role in gut microbiota modulation [111]. Yogurt fat has a particular FA profile enriched in saturated short- and medium-chain fatty acids (SCFA and MCFA), the proportion of which depends on the milk source. Indeed, sheep and goat’s milk contain double amount of SCFA and MCFA, and higher amounts of linoleic acid and conjugated linoleic acid (CLA) compared to cow’s milk [112]. SCFA and MCFA intake has been related to neutral or even beneficial effects on metabolism when consumed at high doses due to their fast metabolization route in contrast to long-chain fatty acids (LCFA), which are transported by chylomicrons to the liver and are easily accumulated in the liver and adipose tissue [113]. The scarce evidence of bacterial changes after MCFA intake comes from an intervention performed with medium-chain triglycerides (MCT) in mice, which resulted in a lower Firmicutes/Bacteroidetes ratio, mainly attributed to reduced *Allobaculum* and *Lachnospiraceae* levels, as well as Proteobacteria levels [114]. In addition, CLA showed a potential prebiotic effect by increasing the *Bacteroidetes/Prevotella* ratio and *Akkermansia muciniphila* levels in mice [115]. However, studies on mice are usually performed with extremely high doses of dietary fats, which obviously do not depict the usual amounts used in the diet for humans. Therefore, further research might consider yogurt as a complete immunomodulatory food, focusing the study on particular bacteria groups with the ability to interact with different yogurt components, such as LAB, fatty acids, and bioactive peptides.

One limitation of the microbiome studies aiming to evaluate probiotics and yogurt effects in healthy adults is the type of sample used (feces) to analyze the gut microbiota. Feces samples are surrogates of colonic samples. Indeed, an increase of probiotic strain levels shows that the strain has passed throughout the colon, probably exerting an effect on the colonic gut microbiota, but not necessarily achieving active colonization. In addition, the precise composition of microorganisms found in stool samples is dependent on the dehydration and fermentation processes present in the rectum, which selects for bacteria that are not found commonly in the lumen [116].

#### 2.2.3. Prebiotics

The prebiotic concept was initially defined in 1995 as a “non-digestible food ingredient that stimulates in a beneficial way the growth and activity of one or a limited number of beneficial bacteria in the gut”. This first definition was modified in 2004 to “selectively fermented ingredients that allow specific changes in both the composition and activity of the gut microbiota, conferring benefits on host well-being and health” [117]. After several modifications, mainly due to discrepancies in the selectivity term, experts of the panel of the International Scientific Association for Probiotics and Prebiotics (ISAPP) defined a prebiotic as “a substrate that is selectively utilized by host microorganisms, conferring a health benefit” [118]. Currently, established prebiotics are carbohydrate-based, but other substances such as polyphenols and polyunsaturated fatty acids (PUFA) converted to the respective conjugated FAs can fit the prebiotic criteria. Selective modulation means that the range of stimulated microorganisms must be limited, and has mainly referred to changes in *Bifidobacteria* and *Lactobacillus*. Further research has suggested that the modulation of other microorganisms with enzymatic machinery to ferment fiber and able to deliver the fiber substrates into the microbial cytoplasm, such as the butyrate-producing bacteria *Fecalibacterium prausnitzii* and *Roseburia* species, can be targets of prebiotic intake, in addition to *Lactobacillus* and *Bifidobacterium* species [117]. Furthermore, the modulatory effect of prebiotics must preferably avoid gas formers such as some *Clostridium* species. Substrates affecting microbiota composition through mechanisms involving selective utilization by host microorganisms are not prebiotics. For instance, fructo-oligosaccharides (FOS) and galacto-oligosaccharides (GOS) are preferentially metabolized by Bifidobacteria [119] through β-fructanosidase and β-galactosidase enzymes, which degrade their linkage bonds [120].

(a) Fibers

According to a recent review by So et al. [121] and the ISAPP Consensus [118], fibers can be classified as: (1) Accepted prebiotic fibers, which include those carbohydrates with substantial scientific evidence of their selective ability to modulate microbial groups, providing a health benefit for the host. This group includes β2-fructans (inulin and FOS), galactans (GOS), lactulose, and human milk oligosaccharides (HMO). (2) Candidate prebiotic fibers, which include polysaccharides with a high potential for prebiotic effects, such as arabinoxylans (AXOS), xylo-oligosaccharides (XOS), and resistant starchs. (3) Dietetic fibers, such as maltodextrins, raffinose, hemicellulose, cellulose, and pectins, among others. Accordingly, evidence of randomized clinical trials is summarized in Table 2a.

The benefits derived from fiber intake relate to its modulatory effect on beneficial bacteria and the reduction of pathogenic or harmful bacteria. Regarding accepted prebiotic fibers, interventions with fructans have revealed an increase in Bifidobacteria taxa compared to the control group [55,56,57,60,61]. Furthermore, the intake of agave inulin at low doses induces profound changes in the gut microbiota, increasing the levels of several species, such as *Bifidobacterium adolescentis*, *Bifidobacterium breve*, *Bifidobacterium longum*, and *Bifidobacterium pseudolongum* [56], as also observed after the consumption of inulin from chicory in *Bifidobacterium fecale/adolescentis, Bifidobacterium longum/breve, Bifidobacterium catenulatum/pseudocatenulatum/kashiwanohense,* and *Bifidobacterium bifidum* [67]. The increase in Bifidobacteria can indirectly affect other bacteria groups, such as butyrate-producing bacteria, due to bacterial cross-feeding mechanisms, which are particularly important in the complex milieu of the GI tract [122]. For instance, *Lachnobacterium, Ruminococcus* [56] and *Coprococcus* levels decrease [58,60], whereas *Anaerostipes hadrus* and *E. rectale* increase [67], suggesting a high variability in the response of the gut micobiota. In addition, fructan intake has also been associated with the reduction of pathogenic or opportunistic bacteria, such as *Desulfovibrio* [56], *Enterobacter*, or *Salmonella* [58]. Scarce evidence exists about GOS effects on healthy adults’ gut microbiota, although an enrichment in *Bifidobacterium* genus, as well as decreases in *Dehalobacterium*, *Synergistes*, and *Holdemania*, have been recently reported after GOS intake [58]. Regarding HMO, which are lactose-based short-chain carbohydrates containing different types of glycosidic bonds, vast evidence exists in infants [123], since they are present in high concentrations in human milk and serve as selective substrates for specific bacteria groups, especially Bifidobacteria [124]. A study in healthy humans revealed that the intake of HMOs, in particular 2′-O-fucosyllactose (2′FL) and lacto-N-neotetraose (LNnT), as well as a mix of both HMOs, can increase Actinobacteria and several Bifidobacteria taxa in a dose-dependent manner (10 or 20 g/d). In particular, the increase in Bifidobacteria seemed to be due to higher levels of *B. adolescentis*. In addition, Firmicutes and Proteobacteria levels decreased over the treatment period compared to basal values, whereas no changes were observed in other beneficial bacteria groups, such as *Lactobacillus* or *F. prausnitzii* [59]. With respect to candidate prebiotic fibers, the intake of resistant maltodextrin and resistant potato starch (RPS) has been also associated with higher levels of *Bifidobacterium* compared to the control group in several studies [62,63,64], whereas *B. ruminantium* [63] and *Bifidobacterium fecale/adolescentis/stercoris* levels increased after the intake of RPS [67]. In addition, some butyrate-producing bacteria, such as *Ruminococcus*, showed different responses to candidate prebiotic fibers depending on the type of fiber source. For instance, higher levels of *R. bromii* were found after the intake of both RPS and resistant starch from maize [63,67], while the levels of *R. obeum* and *R. torques* decreased after RPS intake [63]. Interestingly, XOS and AXOS intake affect Bifidobacteria levels at lower doses than resistant starches (5–10 g/d) [64,65,68], suggesting a stronger prebiotic effect. Recent research on novel fibers, such as polydextrose and soluble corn fiber, has revealed their possible roles as prebiotic fibers [66]. Finally, regarding interventions with dietetic fiber, intake of >80 g/day of whole grains (approximately >26 g fiber/d) over 6 weeks was been related to gut microbiota changes in low consumers of whole grains [69]. However, Tap et al. have reported a dose-dependent effect, showing that the consumption of 40 g/d of fiber from meals in subjects with low species richness can induce a higher microbial change and reduce *E.coli* levels in contrast to the low fiber dose (10 g/d) [21]. In addition, despite the lack of changes in bacteria diversity after the intake of a whole grain diet containing 16 g fiber/1000 kcal during 6 weeks, a reduction of *Enterobacteriaceae* together with an increase of *Lachnospira* were found when compared with a refined low-fiber diet (8 g fiber/1000 kcal) [70].

In summary, the intake of both accepted and candidate fibers in healthy adults positively modulates the levels of Bifidobacteria, suggesting a positive health impact due to their well-known immunomodulary properties. Prebiotic candidate fibers are not officially considered accepted prebiotic fibers, however according to the literature consulted; they can also selectively modulate the levels of *Bifidobacterium* at a higher dose than accepted prebiotics, with the exception of XOS, which can alter the gut microbiota even at a low dose (5–10 g/d). Bifidobacterial genomes have >8% of genes involved in carbohydrate metabolism, and many of them are devoted to the hydrolysis of glycosidic bonds, confirming that Bifidobacteria taxa are able to hydrolyze not only fructans and galactans, but also hemicelluloses, arabinogalactans, AXOS, gums, and branched starches [120]. With respect to *Lactobacillus*, which is considered along with Bifidobacteria as one of the bacteria groups with broader implications in the immunological status of the host, most of the literature consulted employing 16s RNA gene sequencing revealed no changes in this genus [56,58,59]. Only two studies reported increased levels of this genus in feces [61,65], whereas studies using qPCR methodology have not even included *Lactobacillus* quantification in the analysis. The inclusion of this genus in the analysis of the gut microbiota makes sense, since *Lactobacillus* species possess specialized enzymes involved in carbohydrate metabolism, which allow the use of complex carbon sources from fibers [125]. Thus, the design of interventional studies including *Lactobacillus* as one of the bacterial genera analyzed or even focused on *Lactobacillus* species is needed in order to extend the current knowledge about fiber effects on gut and immune health. The high variability in the response of butyrate-producing bacteria and other bacteria groups confirms the complex relationships between bacterial species in the intestinal milieu. On the other hand, the depletion in ammonia and gases producers, such as Proteobacteria or certain *Clostridium* species [126], as well as sulphate-reducing bacteria producers of toxins such as hydrogen sulphide after fiber intake can be beneficial for the GI tract [127]. Furthermore, it is important to note that the extent to which the increase or decrease of certain bacteria groups can affect host health, which might depend on the type of microbial metabolites released by them. For instance, an increase in butyrate-producing bacteria might lead to the release of high amounts of butyrate in the gut, inducing anti-inflammatory effects, but could also promote higher levels of acetic or propionic elements as a result of cross-feeding mechanisms, which have positive impacts on body weight and metabolic outcomes given their roles in satiety modulation and glucose metabolism, respectively [126]. Thus, the existence of cross-feeding mechanisms in the intestinal niche, as well as the different properties of fibers, such as the polymerization degree, which determines fermentability in the gut, can partially explain the differences among studies. In addition, the habitual intake of dietary fiber seems to influence the stability of the gut microbiota in the presence of fiber interventions [60]; thus, changes in dietary fiber intake should be controlled.

(b) Polyphenols

Polyphenols are classified as flavonoids (flavanones, flavones, isoflavons, flavonols, flavan-3-ol, anthocyanidins (ACNs), and proanthocyanidins) and non-flavonoids (hydroxycinnamates, tannins, phenylacetics, benzophenones, xanthones, stilbenes, and lignans). Of these, 90%–95% polyphenols are not absorbed in the small intestine and reach the colon, where they undergo an extensive breakdown into low molecular weight phenolic metabolites by a wide range of microbial species [128,129]. In particular, gut transformation of dietary polyphenols depends on their chemical structure, especially the type of initial glycosylation pattern, which will determine their absorption and bioavailability [130]. Most evidence about polyphenol effects on the gut microbiota come from in vitro and animal studies. Scarce research has been conducted on healthy adults, with most being interventional studies performed with a small number of subjects with complete foods (fruits, vegetables, cocoa, or tea) instead of specific phenolic compounds (Table 2b). In this respect, there is some controversy regarding interventional trials with fruits and vegetables. Indeed, while no changes in the gut microbiota composition were shown after 4 weeks consumption of a boysenberry juice with high amounts of ACNs and ellagic acid [73], in another study the intake of a wild blueberry drink enriched with ACNs induced an increase in *Bifidobacterium* levels [71], and in particular *B. longum* spp. *infantis* [72]. These findings were partially expected, given the ability of Bifidobacteria to metabolize the sugar moiety of ACNs to obtain carbon and energy when they are present as glycosylated molecules, as observed in fruits [131]. In addition, a progressive increase in fruit and vegetable intake across 18 weeks of intervention induced more gut microbiota changes in the low flavonoid group than in the high flavonoid group, promoting higher levels of *Bifidobacterium*, *Bacteroides/Prevotella*, *C. leptum*, and *R. bromii*. Surprisingly, the intake of total flavonoids from fruits and vegetables was lower compared to the high flavonoid group, suggesting that polyphenols from other food sources such as tea or cacao, as well as the fiber content of the diets, might have driven the observed changes [74]. The intake of a high cocoa drink increased *Bifidobacterium* and *Lactobacillus*–*Enterococcus* levels compared to a low cocoa drink, despite both drinks containing the same fiber content, thus suggesting that changes could be mediated by cocoa flavonols [75]. The increase in *Lactobacillus* levels in this study is noteworthy, since most studies with prebiotics have not shown changes in this bacteria group, as mentioned above. In addition, the high cocoa drink also induced a decrease in the *C. histolyticum* group, which includes some pathogenic species such as *C. perfringens*, revealing a positive role of cocoa flavonols in gut health. With respect to green tea polyphenols, one study performed with 12 healthy adults showed robust changes in the gut microbiota composition, increasing both genus and species α-diversity, as well as in different bacteria families and in Firmicutes and Actinobacteria genera [76]. However, no changes were observed in the main phyla analyzed after green tea intake in another study with a bigger sample size (*n* = 58) [77].

Overall, the effects of polyphenols on the gut microbiota seem to be similar to those found with accepted prebiotic fibers, increasing the *Bifidobacterium* levels, which is partially attributed to their sugar-enriched chemical structure. However, more research is needed to ascertain the roles of specific phenolic compounds in isolated supplementation protocols to avoid the masked effect of confounder factors, such as the dietary fiber present in the tested polyphenol-enriched foods and other habitually consumed foods.

#### 2.2.4. Alcoholic Beverages

The detrimental effects of alcohol on health re widely known. Alcohol abuse is related to several pathologies of inflammatory condition, such as liver, intestinal, and mental diseases [132,133]. One of the proposed mechanisms relating alcohol intake with the development of inflammatory diseases is the promotion of gut dysbiosis. Indeed, gut microbiota of alcoholics is characterized by an enrichment of Proteobacteria and a depletion of Bacteroidete levels compared to healthy controls [134]. Another typical feature of alcoholic subjects is the increased levels of plasma endotoxin (LPS) and proinflammatory cytokines, which reflects damage to the intestinal barrier [134,135]. In this context, Leclerq et al. showed that intestinal permeability (IP) mediates the interactions between the lumen gut microbiota and the immune cells of the lamina propia, promoting the development of anti-inflammatory or proinflammatory responses. Indeed, alcoholic subjects with high permeability showed large decreases in the overall bacterial load and the *Ruminococcaceae* family, as well as higher levels of *Lachnospiraceae* and *Blautia* compared to alcoholics with a low IP and healthy controls. The dysbiotic group included both actively drinking and sober alcoholics (>1 month), suggesting that dysbiosis is maintained over the long term. In addition, *Lactobacillus* and *Bifidobacterium*, as well as *Ruminococcaceae* species, increased during alcohol abstinence, suggesting their possible roles in the recovery of IP. The authors hypothesized that metabolites derived from gut proteolytic fermentation might originate from gut barrier dysfunction and inflammation, involving branched-chain FAs, indolic compounds, and potentially toxic metabolites, such as phenolic and sulfur-containing compounds [136].

As mentioned above, most human studies have been conducted on alcohol abuse, whereas evidence of the effects of moderate consumption of alcohol on health is mainly derived from epidemiological studies. In this sense, a moderate consumption of alcohol (up to one drink a day for women and up to two for men) has not been associated with detrimental effects on health in some studies [137], and even with neutral or beneficial effects on cardiovascular health and diabetes [138,139]. These findings reveal the possibility that other components of alcoholic drinks could play roles in this sense, such as polyphenols or prebiotic fibers. For instance, distilled alcoholic beverages exclusively contain high amounts of alcohol (around 40% of alcoholic degree), while fermented alcoholic drinks are rich in beneficial nutrients, such as polyphenols and fibers, and contain a lower dose of ethanol compared to distilled drinks (around 4%–8% for beer and 11%–14% for wine). Beer consists of the prebiotic fibers XO and AXOS [140], and also contains a rich profile of polyphenols (catechins, phenolic acids (e.g., ferulic acid), and flavonoids (e.g., xanthohumol)) [141]. On the other hand, red wine is one of the most polyphenol-enriched beverages, containing a high amount and variety of polyphenols, such as ACNs, flavan-3-ols, non-flavonoids (stilbenes), gallic acid, proanthocyanins, catechins, and phenolic acids [142], and is the most studied beverage in healthy adults (Table 3). Regarding observational studies, the intake of red wine was associated with lower levels of the beneficial bacteria *Bifidobacterium, Blautia coccoides, C. leptum*, and *Lactobacillus* compared to non-consumers of red wine [78]. Despite the authors relating these findings to the antimicrobial effect of wine polyphenols, this would be more plausible if reduced levels of common pathogens such as *E. coli* or *Salmonella* had been observed [143]. The roles of other dietary polyphenols, such as those found in fruits and vegetables (ACNs, flavonols, and hydroxybenzoic acids), together with the higher intake of ethanol consumed, could affect the observed changes [78]. Another observational study with a single dose of vodka revealed no changes in either the main bacteria groups analyzed nor in diversity indexes measured in the 4 h after consumption, suggesting that a single portion of ethanol is not sufficient to alter the microbiome [79]. Regarding interventional studies, exclusive ethanol intake with gin over 4 weeks promoted an increase in *Clostridium* and *C. histolyticum* groups compared to both red wine and dealcoholized red wine. Despite no differences being observed between red wine interventions, the intake of red wine induced an overall greater impact on gut bacterial groups, as shown by the higher levels of *Prevotella, Bifidobacterium, Blautia-coccoides, Enterococcus, Bacteroides*, and *Eggerthella lenta*, suggesting that both polyphenol and ethanol intake might be responsible for such changes [80]. On the contrary, Barroso et al. failed to show changes in the levels of main bacterial groups after the intake of red wine over one month, despite increased α-diversity values, as well as increased levels of minor genera with a documented capability to metabolize polyphenols, such as *Slackia, Gordonibacter, Oscillatoria*, and *Veillonella* [81]. The only interventional study investigating the effects of beer on the gut microbiota of healthy adults found that non-alcoholic beer changed the gut microbiota composition to a greater extent than alcoholic beer. In particular, the α-diversity and the levels of Bacteroidetes, Actinobacteria, and several bacterial genera were higher after the intake of non-alcoholic beer [82], suggesting that the absence of ethanol and the different phenolic profiles present in non-alcoholic beer might be responsible for such changes [144].

In summary, it seems that the three key components of alcoholic fermented beverages, namely polyphenols, fibers, and ethanol, exert a synergic effect on the gut microbiota. However, the impact of ethanol itself on the gut microbiota has not been sufficiently investigated. Reasons to extend ethanol research in healthy adults include the evidence of different gut microbiota responses to non-alcoholic red wine and non-alcoholic beer compared to their alcoholic forms, and the existence of bacteria species capable of metabolizing ethanol, such as the *Enterobacteriaceae* family [145]. In addition, future research might extend the knowledge on gut microbiota changes due to the intake of non-alcoholic beer and alcoholic beer, given their high consumption in the habitual diet.

#### 2.2.5. Refined Sugars and Sweeteners

Refined sugars are, along with refined fats, primary ingredients of processed foods characteristic of Western diets. The consumption of Western diets has been related to the development of obesity because of their detrimental roles in glucose and insulin metabolism, and consequently in fat deposition, and to lower bacteria diversity, even to a greater extent than the effects from high BMI values [146]. Gut microbes modulate glucose absorption and its flow to the liver, consequently playing key roles in insulin regulation [147]. Indeed, gut dysbiosis is a typical feature of obesity and type 2 diabetes [148,149]. Sucrose, or common sugar, is a disaccharide composed of fructose and glucose, which is mainly absorbed in the small intestine, but it can also be metabolized by some gut microbial species [150]. Indeed, unabsorbed or malabsorbed sucrose derived from excessive dietary intake can reach the colon and act as a substrate for microbiota metabolism, which must deal with the excessive loads of these familiar substrates (fructose) and also adapt to “unfamiliar” substrates, such as sugar alcohols or certain artificial sweeteners [150]. Fructose intake has increased in recent years as a substitute for sucrose in processed foods in the form of high-fructose corn syrup, mainly due to its neutral effects on glucose levels. However, an excessive intake of fructose seems to be detrimental to the liver due to its effects on the gut microbiota by promoting the increase of proinflammatory bacteria, endotoxin levels, and the loss of tight junction proteins, leading to higher expression of TLRs in the liver and proinflammatory cytokines [151,152]. In this respect, studies on mice have confirmed the detrimental effects of high fructose diets by inducing gut dysbiosis. For instance, high amounts of fructose in a high-fat diet model reduced Bacteroidete and increased Firmicute levels compared to a control group consuming only sucrose [152]. However, the opposite was observed by Ferrere et al., reporting higher levels of *Bacteroides* and *Erysipelotrichi* in the group consuming fructose as part of a control diet [153], despite these differences being partially caused by the type of fat-enriched diet administered. The scarce evidence from human studies evaluating sucrose or fructose effects comes mainly from research on Western diets, revealing lower bacteria diversity related to refined-sugar-enriched diets [146].

A common strategy employed to reduce sugar intake and the incidence of obesity is the use of sugar alcohols (polyols), along with natural (stevia) and artificial sweeteners (aspartame, saccharine, acesulfame-K, and cyclamates) [154]. However, the fact that the obesity epidemic has also increased dramatically in parallel to the increase in artificial sweetener intake weakens the hypothesis that they could prevent the development of obesity [155]. In fact, studies on mice have revealed that the intake of artificial sweeteners has a detrimental effects on metabolism, as observed in supplementation protocols with saccharine leading to a greater extent of glucose intolerance than oral glucose in a mechanism mediated by changes in the gut microbiota [86]. However, the dose of saccharine was extremely high compared to the admissible daily intake (ADI) for humans, suggesting the need for appropriate clinical trials in humans in order to confirm these findings. In this respect, there is scarce information about the impact of sweeteners in the context of the habitual diet. Observational studies have revealed a different bacterial diversity in aspartame and acesulfame-K consumers compared to non-consumers, mainly due to differences in low-abundance bacteria [85]. In addition, the intake of non-caloric artificial sweeteners (NAS) in healthy adults was positively related to *Enterobacteriaceae* and Actinobacteria levels in an observational study [86]. These findings led to an intervention being carried out with saccharine in non-diabetic subjects and non-consumers of NAS. In this study, the subjects were divided into NAS responders, who developed glucose intolerance after 6 days of saccharine consumption, and non-NAS responders. The microbiomes were clustered differently between NAS responders and non-responders, and NAS responders showed higher levels of Lactobacillales and Bacteroidales but lower of Clostridiales at the end of the treatment compared to basal values, probably due to differences in the basal microbiota composition. Furthermore, the fecal transplant of NAS responders to germ-free mice induced similar changes, inducing higher levels of *Bacteroides fragilis* and *Wisella cibaria* (Lactobacillales order) and lower levels of *Candidatus Arthromitus* (Clostridiales) [86]. With respect to polyols, interventional studies have revealed potential prebiotic effects of isomalt and lactitol by increasing Bifidobacteria levels [84]. In addition, the intake of isomalt induced an increase in *Atopobium* levels and decreases in *Roseburia intestinalis* and *Bacteroides* levels compared to sucrose intake (Table 4) [83]. 

In summary, research into the isolated effects of sucrose and fructose in humans is very scarce, which seems to be reasonable due to the negative effects on health associated with the intake of these sugars. The evidence from animal studies about the ability of some bacteria species to metabolize fructose suggests that excessive amounts of fructose in the human diet can change the gut microbiota and mediate the inflammatory response observed in non-alcoholic liver disease [153]. The massive use of sweeteners as sugar substitutes has made the study of their effects on gut health necessary, considering that each sweetener has a particular chemical structure, and thus a different metabolization route. For instance, stevia glycosides and polyols are mostly metabolized in the colon, but aspartame and cyclamate are totally hydrolyzed in the duodenum [156]; thus, gut microbiota changes should not always be expected after the intake of sweetener. Future research in this field should investigate the effect of sweeteners with the common doses found in the habitual diet, since most current studies in animal models employ extremely high doses compared to the admissible daily intake (ADI) level, meaning the results cannot be extrapolated to humans (Table 4).

#### 2.2.6. Fats

Germ-free mice models and gut microbiota transplants are some of the strategies providing new insights about the roles and mechanisms through which the gut microbiota impact host metabolism. In this respect, fecal transplants from obese adults consumers of fat-enriched diets helped increase the understanding of the key roles of gut microbes on adiposity and metabolic outcomes [157], similar to what was observed in conventional mice subjected to high-fat diets [158,159]. These models confirmed the close connection between the intake of dietary fats and the gut microbiota. Later, the discovery that high amounts of fats were able to induce gut dysbiosis even before the onset of obesity brought more clarity in this respect. Indeed, bacterial changes can be a consequence of the metabolic disturbances caused by excessive loads of lipids in the blood or by the arrival of unusually high amounts of fat to the colon [160]. Most dietary fat interventions have been performed in animal populations with extremely high doses of fats exceeding the typical amounts usually found in human diets. On the other hand, the scarce evidence in humans comes from supplementation protocols with specific FAs and interventions with increased amounts of fat in the context of the habitual diet (Table 5), with the latter often considered controversial due to the negative relationship between high fat intake and health outcomes [161]. In this sense, increased intake of LCFAs in the form of dairy products or butter induces changes in a vast number of bacterial genera compared to a low saturated fat diet [88]. The effects of different amounts of soybean oil were also investigated in healthy adults, revealing lower levels of Firmicutes, *Blautia*, and *Fecalibacterium*, and higher levels of Bacteroidetes, *Alistipes*, and *Bacteroides* in the high-fat group compared to the low-fat group [87]. The high contribution of omega-6 PUFAs in the high-fat group, which was previously related to proinflammatory effects [162], and the lower amount of whole carbohydrates might explain the microbial changes observed. Regarding isolated supplementation with a specific dose of FAs, the intake of the omega-3 PUFAs, such as docosahexaenoic acid (DHA) plus eicosapentaenoic acid (EPA), over 2 weeks promoted an increase in beneficial bacteria, such as *Bifidobacterium*, *Lactobacillus*, *Lachnospira*, and *Roseburia*, but decreased *Fecalibacterium*, which is also considered a positive bacteria for gut health due to its anti-inflammatory effects [89]. Therefore, the modulation of some beneficial bacteria groups and not others might suggest the existence of cross-feeding mechanisms and confirm the influence of the initial gut microbiota composition before intervention in modulating the response to diet. In addition, the intake of a highly amount of dairy cream (source of LCFA) over 7 days did not affect bacterial diversity but did reduce Bacteroidetes and increased β-Proteobacteria levels [90]. As mentioned above, both the quantity and the quality of fat are relevant. Comparative studies with different types of fats have mainly been performed in mice populations [163]. However, a recent study conducted in our lab in apparently healthy adults with borderline high levels of plasma cholesterol revealed that the intake of yogurts with different FA profiles affected certain bacteria groups in subjects with the highest total cholesterol/high-density lipoprotein (HDL)–cho ratio. Specifically, the intake of whole ewe’s milk yogurt over 5 weeks, which differs from semi-skimmed (ES) ewe’s yogurt and from cow’s milk yogurt in fat quality, decreased *Blautia-coccoides* only in women of this group when compared to the period of ES yogurt intake. In addition, cow’s milk yogurt increased *C. leptum* compared to ES only in subjects of the medium- and low-cholesterol/HDL–cho ratio [91].

The mechanisms by which dietary fats affect the gut microbiota composition and functionality have still not been elucidated. Most dietary fats are digested by intestinal lipases and absorbed in the small intestine, but recent evidence has revealed that with normal consumption of dietary fat, a small proportion of the resulting free FAs (7%) escapes the small intestine and reaches the colon, where FAs can interact with bacteria groups [164]. The metabolization of dietary fat requires oxygen and the gut microbiota is dominated by strict anaerobes, complicating the use of fat as an energy source for gut bacteria [111]. In addition, high-fat diets are usually accompanied by low amounts of carbohydrates and fiber, which restricts the availability of fermentation substrates for bacteria. The proposed mechanisms by which dietary fats affect the gut microbiota are a bactericidal effect on cell membranes, impairing intracellular metabolism and decreasing the bacterial load [165], and the modification of bile acid metabolism through the action of bacterial biliary hydrolases, which are important for lipid digestion and absorption [166]. Lower levels of bacteria involved in bile acid metabolism can damage the metabolic processes that depend on it, such as cholesterol metabolism, damaging the host metabolism [167]. As mentioned above, the different chemical structures, and in particular, the length of FA chain determine the absorption and metabolization, and thus the effects on health. In particular, MCFAs are not incorporated in triglycerides, since they are directly transported to the liver via the portal vein, where they undergo β-oxidation. In contrast, LCFAs are transported into chylomicrons in the systemic circulation through the thoracic ducts, reaching the liver and easily accumulating in the adipose tissue [113]. In addition, they increase the levels of plasma LPS from the cell wall of Gram-negative bacteria, promoting “metabolic endotoxemia”. They then settle in target tissues, such as adipose tissue, where they join CD14 (a marker of innate immune cells) and promote a proinflammatory state [168].

In conclusion, the metabolic benefits derived from the intake of omega-3 PUFAs, MUFAs, as well as saturated SCFAs and MCFAs, together with the prebiotic effects demonstrated in animal studies [163], motivate the design of appropriate interventional studies aimed at investigating their effects in healthy adults. In addition, the increased intake of fats in the diet worldwide [169] makes fat a new and possible substrate in our gut microbiota.

## 3. Stress

The word stress has an ambiguous meaning, since it is used in different situations in life and can have a positive or negative connotation. In general, “good stress” refers to a situation involving a risk, with a feeling of being rewarded by a positive outcome, such as a salary rise. In addition, stress can also be tolerable if, despite a bad situation happening, the individual is able to cope with it due to having a positive, adaptive, healthy mind. However, “bad stress” or “toxic stress” refers to unpleasant situations that cannot be faced by the individual due to having poor support and brain architecture. In this case, stress can be mainly related to early life events that impair the development of good impulse control and adequate self-esteem. In this situation, the inability to cope with stress can promote adverse effects on behaviour and physiology, and in turn on health maintenance [170]. In this sense, acute and chronic stress have opposite health effects, ranging from healthy activation of the immune system to immunosuppression, respectively. Therefore, stress not only affects the brain, but also affects the immune system and the GI tract, among others, via the brain–gut microbiota axis [171]. The first evidence that gut microbiota affect neural development again came from germ-free mice models, which showed an exaggerated response to stress. This study helped in understanding the key roles that gut microbes play in neuronal circuits involved in motor control, anxiety, and social responses [172,173]. Through the production of neurotransmitters and SCFAs and the stimulation of cytokine release from immune cells, the gut microbiota affect brain function via the vagus nerve. In a similar way, the brain influences the gut microbiota composition through the same pathway and also via the hypothalamus–pituitary–adrenal axis [171]. Therefore, an external stressful stimulus can have bidirectional effects on both the gut microbiota and brain function. Stressful determinants can be physiological and psychological. Physiological stress includes the practice of extreme physical activity, such as military training. Indeed, this kind of training involves not only prolonged physical activity but also dealing with extreme temperatures (hot or cold), sleep deprivation, and psychological stress due to the strict discipline demanded by the military. The consequences in the GI tract can be detrimental and involve damage to intestinal barrier permeability, inflammation, and dysbiosis [174]. On the other hand, sleep deprivation is known to be a stressful condition for the human body. Indeed, it involves the increase in certain proinflammatory cytokines, such as IL-6 and IL-1β [175], and circadian rhythm disruption, which in turn affects the activity of several hormones [176] and gut microorganisms [177]. The limited scientific evidence in healthy humans has revealed higher levels of *Coriobacteriaceae* and *Erysipelotrichaceae* (Actinobacteria and Firmicutes phyla, respectively), even after brief deprivation of sleep for 48 h sleep (only 4 h of sleep/day) [178]. In addition, good quality of sleep has been associated with higher levels of bacteria belonging to Verrucomicrobia and *Lentisphaerae* phyla, as well as improved performance in cognitive tasks. Smith et al. confirmed the role of IL-6 in the sleep–microbiome relationship, as well as a positive correlation between bacterial α-diversity and Actinobacteria levels, in line with Benedict et al., although it is important to consider the observational nature of both studies. The precise mechanisms involved in the effects of sleep on the gut microbiota are still not well understood, but the identification of microbial metabolites that interface through the brain–gut microbiota axis could provide more insights in this respect. For instance, γ-aminobutyric acid (GABA) and serotonin are key neurotransmitters involved in the process of sleep [179,180] and are synthetized by different bacterial species [181,182]. More research exists on obesity with regard to sleep deprivation effects. In particular, obesity is associated with sleep alterations and poor dietary habits characterized by unusual feeding times and later chronotypes, with a shift in sleep–wake timing towards eveningness [176]. Consequently, there is a disruption of the circadian clock [183], which regulates energy utilization [184], and changes in appetite, regulating hormones such as leptin and ghrelin, which in turn might affect dietary choices and body weight [176]. In particular, evening chronotypes are more likely to consume larger portion sizes, second rounds, and energy-rich foods, as well as a higher emotional eating score [185] and decreased resting energy expenditure, and glucose tolerance [186]. Therefore, a connection seems plausible between an altered microbial profile, sleep deprivation, and the dietary changes associated with obesity.

Regarding psychological stress, the high prevalence of intestinal disorders, such as irritable bowel syndrome (IBS), found in mental diseases, such as depression [187], as well as the proven efficacy of specific probiotics strains in the improvement of stress symptoms and anxiety (known as psychobiotics) have confirmed the close connection between the gut microbiota and the brain in disease states [188]. Emotional distress states, such as anxiety and depression, are features of mood and personality disorders, which are also linked to increased GI symptoms and changes in the gut microbiota of healthy adults. For instance, one study investigating the relationship among diet, gut microbiota, and mood disorders revealed a sex-dependent association. *Lactobacillus* was inversely associated with depression scores among males, whereas *Bifidobacterium* showed an inverse relationship with anxiety scores among females [188]. Interestingly, *Lactobacillus* spp. and *Bifidobacterium* spp. are considered psychobiotic species due to their mood enhancement properties and their positive influence on the brain–gut microbiota axis [189]. In addition, *Peptostreptococcaeae* levels also increased alongside anxiety symptoms, in agreement with the study by Kim et al., which showed a positive correlation between *Peptostreptococaceae* and *Gammaproteobacteria* with neuroticism, a personality trait associated with an increased risk of anxiety disorders. Furthermore, low conscientiousness was positively correlated with Proteobacteria levels [190]. However, the cross-sectional nature of these studies makes it difficult to establish a causal relationship. The effects of negative emotions on health go beyond the GI tract, affecting the immune system and brain function, as observed in the study by Sutin et al., which revealed a link between neuroticism and low conscientiousness with chronic inflammation and the activation of the hypothalamic–pituitary–adrenocortical (HPA) axis [191]. In addition, the growing evidence revealing a sex-specific pattern in gut microbiota–brain interactions [192] and the fact that emotional disorders are dramatically increasing [193] make the design of appropriate interventional studies employing psychological therapy, appropriate mental health questionnaires, and metagenomic approaches necessary, in order to ascertain the precise mechanisms involved and to improve the quality of life of apparently healthy men and women without mental pathologies but with high emotional distress.

## 4. Other Lifestyle Factors

Besides diet and stress, recent literature has pointed out the relationship between the gut microbiota and frequent but often forgotten lifestyle factors, such as physical activity, drug consumption, place of living, and tobacco habits.

### 4.1. Physical Activity

There is scientific evidence on the health-promoting effect that exercise has in humans, which could be partially attributed to the modulation of the gut microbiota composition. Indeed, active subjects such as rugby players have a healthier gut microbiota composition compared to sedentary subjects; in particular, higher α-diversity and *Akkermansia* levels. The values of α-diversity were related to protein intake, which suggests that both diet and active physical activity are drivers of gut microbiota changes [194]. Likewise, active women have shown higher levels of some beneficial bacteria (*F. prausnitzii*, *Roseburia hominis*, and *A. muciniphila*) compared to sedentary women [195]. However, the effect of exercise alone on intestinal microbiota independently of the dietary habits, which may have a greater impact on intestinal microbiota than exercise, has been poorly examined. For this purpose, Cronin et al. investigated microbiome changes in physically inactive individuals after an intervention of 8 weeks with moderate exercise and protein supplementation. The main finding was the lack of changes in bacterial diversity and the levels of the bacterial taxa analyzed, suggesting that moderate exercise does not exert an effect on untrained subjects [196]. These results are contrary to what had been observed in active subjects, suggesting that the intensity of the exercise impacts the gut microbiota in a different way. Indeed, elite athletes seem to have a metabolically favorable intestinal microbiome as a manifestation of many years of optimized nutrition and a high degree of physical condition throughout the years [197]. The mechanisms by which moderate exercise might affect gut communities involve the association of moderate exercise with a lesser degree of IP, the preservation of mucous thickness, lower rates of bacterial translocation, and the upregulation of the production of antimicrobial proteins, such as defensins [198]. On the other hand, some authors have proposed the existence of a muscle–microbiota axis, since the muscles express TLR-4 and TLR-5 receptors, which can be activated by circulating LPS from gut bacteria and can stimulate the production of inflammatory cytokines in the muscle. In addition, the practice of moderate physical activity can modulate bacterial functions, as observed by the higher levels of butyrate, which in turn participate in the regulation of cholesterol, glucose, and lipids in the muscle. Furthermore, moderate exercise can also stimulate the production of gut IgA and the levels of certain lymphocyte populations, as well as reduce the intestinal transit time; all these mechanisms might influence the gut microbiota composition [199].

### 4.2. Drug and Air Pollutants

Drug intake, together with diet, is one of the most relevant factors involved in gut microbiota changes [200]. The study by Falony et al. revealed that medication exposure was the main factor causing the greatest variability in the gut microbiota composition in 1016 healthy adults [201]. However, this study was many years ago, following the discovery that antibiotics can abolish a broad range of gut bacteria groups, which led to the design of antibiotic-treated mice models in order to identify key roles of gut microbes on human health [202]. Indeed, the most common finding after antibiotic intake is the decrease in bacterial diversity, which depends on the type of antibiotic administered [203]. Despite the cessation of antibiotic use normally inducing the recovery of the basal gut microbiota composition in the short term, some permanent changes can occur, such as a lower resistance to pathogens colonization, which in turn increase the infection risk and the susceptibility to disease development [200]. Other types of drugs such as antihistamines, statins, or mucolytic agents can also affect the gut microbiota composition. In addition, polypharmacy or the co-administration of different drugs may promote an abundance of microbial taxa that can metabolize different types of drugs [204]. Gut bacteria have a broad enzymatic ability to directly metabolize drugs, mainly by reduction and hydrolysis reactions. The most studied enzyme is β-glucuronidase, which is present in many different bacteria groups, such as *Clostridium, Streptococcus, Lactobacillus, Ruminococcus*, and *Bifidobacterium* [205], whose main role is to remove 50% of glucuronic acid from hepatic phase 2 metabolites, as observed after the consumption of irinotecan (SN-38 glucuronide) and nonsteroidal anti-inflammatory drug (NSAIDS) [206]. In addition, some microbial metabolites such as SCFAs can act in the host capability to metabolize drugs. Furthermore, they can indirectly affect the host metabolization of drugs by influencing hepatic function, since many microbial-derived metabolites can compete with drug intermediates of hepatic metabolic reactions, and thereby interfere with host detoxification pathways, as observed with paracetamol and the microbial metabolite P-cresol [207]. On the other hand, drugs can affect the gut microbiota by exerting antibacterial activity, changing the GI tract environment (e.g., pH and transit time), mucosa integrity, host and bacterial metabolic activity, and the production of microbial metabolites [200].

The choice of the place of living can also influence our gut microbiota. Indeed, the impact of air pollution on host health goes beyond heart and lung health. Air pollutants consist of a complex mixture of different compounds, including gases and particulate matter, as well as the microbes suspended with them. They can be ingested in food and water containing such particles or after mucociliary transport mechanisms that expel them from the lungs following deposition during inhalation [208]. Indeed, gut microbiota and the GI tract can be affected by air pollutants. In particular, some air pollutants have been related to GI diseases, such as inflammatory bowel diseases [209], but the underlying mechanisms are still unknown, although inflammation is a likely cause. The conclusions derived from a recent systematic review revealed a mild alteration in the gut microbiota, which was mainly derived from mice studies. Future research in humans is warranted, as well as more advanced community sequencing technologies and complete compositional analysis of the particulate matter and gases to which humans are exposed [208].

In conclusion, the extremely high intake of drugs worldwide, not only by old people or patients, but also by supposedly healthy adults, the worsening of air quality due to urbanization processes, and the novel knowledge about the underlying mechanisms between drugs and gut microbiota and its impact on host metabolism mean drug consumption and air quality are two relevant factors in microbiome human studies.

### 4.3. Tobacco Consumption

Despite tobacco consumption seemingly having no effect on the gut microbiota, the evidence that smoker patients with Crohn’s disease showed lower bacterial diversity and reduced levels of *Collinsella, Enterorhabdus*, and *Gordonibacter* compared to non-smoker patients [210], calling into question the possible relationship between smoking and the gut microbiota. Furthermore, despite the scarcity of evidence in healthy adults, cessation of smoking for 8 weeks can modify the gut microbiota of healthy adults, increasing bacterial diversity and Firmicutes and Actinobacteria levels, and decreasing Proteobacteria and Bacteroidetes levels [211]. Despite diet being controlled and not being related to microbiome changes, the small sample size and the role of other potential factors that were not controlled could limit the power of these findings. The mechanisms that could relate tobacco consumption to changes in the gut microbiota are not well-known, but changes in mucosal immunity and IP have been suggested as some of the involved pathways [211]. The increased consumption of tobacco from the earliest ages, together with well-known deleterious effects of tobacco in the progression of intestinal diseases [212], makes this an important field of research related to the microbiome.

## 5. Key Factors Involved in the Diet–Gut Microbiota Interaction

**Figure 3:**
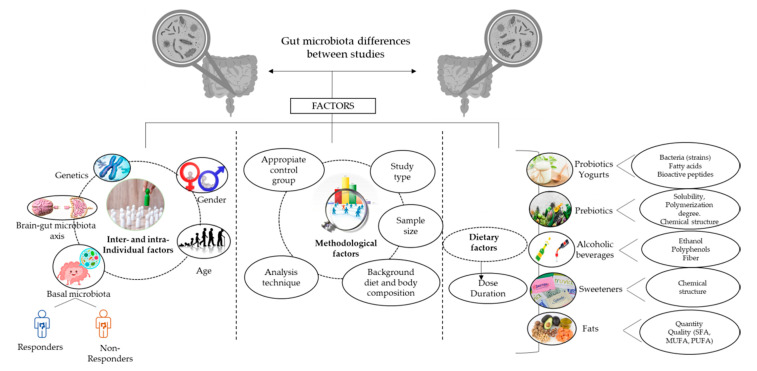
Relevant factors to consider when interpreting results derived from microbiome-based studies. Several factors must be considered in the interpretation of gut microbiota findings among studies. Firstly, the inter-individual factors, including demographic factors such as age and gender, and intra-individual factors, including the intrinsic characteristics of an individual, must be considered, which influence the response of the gut microbiota to a dietary intervention (responders and non-responders), such as genetics, the functionality of the brain–gut microbiota axis, and the basal microbiota composition. Secondly, methodological factors include the existence of an appropriate control group, the nature of the study (observational or interventional), the microbiota analysis technique (culture, qPCR, FISH, or 16s RNA gene sequencing methods), and the control of the background diet and body weight. Finally, factors related to the dietary intervention must be considered, highlighting the dose and duration of the treatment as common aspects that should be considered. SFA: Saturated fatty acids; MUFA; Monounsaturated fatty acids; PUFA: polyunsaturated fatty acids.

Data interpretation is one of the trickiest steps when comparing results between studies. One of the reasons is the wide number of inter-individual and intra-individual factors that can influence the obtained microbial data, as described below (Figure 3).

### 5.1. Intra-Individual Factors

Recent evidence has pointed out that intra-individual variability explains gut microbiota changes [213] to a greater extent than diet does [88]. Intra-subject factors involve the ability of the internal organism processes to maintain the homeostasis and resilience of the host in the adaptation to external stimuli, such as stressful conditions. In this situation, poor activity of the nervous system will cause immunosuppression and sleep disruption, affecting hormone levels and the gut microbiota composition and functionality, in addition to altering the responses of gut microbes to diet. In this sense, the initial bacterial richness before the beginning of an intervention seems to be a determinant in the response of the gut microbiota to diet [88].

### 5.2. Inter-Individual Factors

#### 5.2.1. Geography

**Figure 4:**
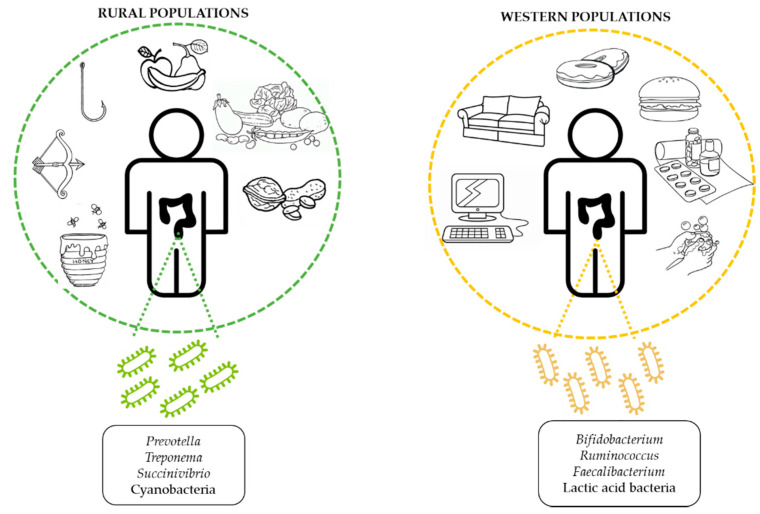
Bacterial profiles and lifestyle factors for rural and urban populations. The geographical area is one of the main drivers of the differences in microbial diversity between populations across the world. On one hand, a large intake of natural products rich in fiber predominates in rural populations, leading to higher levels of taxa involved in fiber processing, such as Prevotella or Treponema. In addition, some opportunistic bacteria such as Succinivibrio and Cyanobacteria are more present in rural populations. Both diet and physical activity influence the different microbiota profiles, including the practice of harvesting, hunting, and fishing. In contrast, urban populations tend to have a less healthy lifestyle characterized by diets high in refined fats and sugars, sedentarism, high intake of drugs, and extreme hygiene, characterized by a gut microbiota enriched in species of *Bifidobacterium*, *Ruminococcus*, *Fecalibacterium*, and acid-lactic bacteria.

Geographic factors include genetic and cultural factors of the different populations across the world and represent one of the main inter-individual factors responsible for the differences in microbial diversity among rural and urban populations [29]. One of the most relevant findings is the lower bacterial diversity and the loss of bacterial taxa involved in fiber processing in urban populations [214], including American and European cohorts, but also African populations with a more Western lifestyle, such as the African “Bantu” population. Metagenomic studies have revealed a different bacterial profile in the Bantu people compared to the BaAka hunter–gatherer population (also African), suggesting a gradient of subsistence. Indeed, the BaAka are consumers of high amounts of fibrous starches and leaves, thus probably influencing the enrichment of fibrolytic bacterial taxa, such as *Prevotella* or *Treponema*, found in their gut microbiota, in addition to unclassified *Clostridiaceae* and Cyanobacteria. This outcome could suggest that a transition from a traditional to modern agricultural Western-like lifestyle results in a gradual decline in such microbes. On the other hand, the higher levels of *Fecalibacterium* and LAB in the Bantu may reflect a higher availability of digestible sugars. Despite these differences, the gut microbes of BaAka and the Bantu were more similar to each other than to that of Westerners [29]. Similarly, the gut microbiota of the Hadza hunter–gatherers from Tanzania showed a similar microbiota profile, which was enriched in *Treponema, Succinivibrio*, and *Prevotella*, and deficient in *Fecalibacterium, Ruminococcus*, and *Bifidobacterium* compared to an Italian urban population. Indeed, the Hadza showed an unusual arrangement of unclassified Clostridiales and a general reduction in butyrate producers, such as *Clostridium* cluster IV and XIV. The enrichment of opportunistic microbes such as Proteobacteria, *Succinivibrio*, and *Treponema* could be considered rare, since they can act as pathogens under certain circumstances, however they fortunately provide beneficial functions for host health. For instance, some *Treponema* species such as cellulose and xylans hydrolyzers may help to extract nutrients from fibrous foods, which are part of the traditional African diet [215]. Some authors have hypothesized that the absence of Bifidobacteria in Hadza populations could be due to the lack of dairy products, as supported in other studies carried out in Koreans and vegans [216,217]. The fact that Bifidobacteria were nearly absent in rural populations, as well as the enrichment in “opportunistic” bacteria of Proteobacteria and Spirochaetes phyla, reflects the necessity to re-evaluate the standards for “healthy” and “unhealthy” gut microbiota depending on the geographic context (Figure 4) [218]. 

#### 5.2.2. Gender

The different dietary responses found in women compared to men in several studies, together with the fact that the prevalence of some immune-related diseases differs between sexes, has increased the interest in studying sex as an independent immunomodulatory factor in microbiome studies. As examples, female participants showed larger shifts in the abundance of *Bifidobacterium* after the intake of inulin compared to males [56], in agreement with the study by Bédard et al., which showed a gender-dependent response in lipids and inflammatory biomarkers after a Mediterranean diet intervention [219]. In addition, while the prevalence of infections is high in men, women usually are more predisposed to autoimmune disease development [220,221], such as type 1 diabetes [222] or rheumatoid arthritis [223].

The potential factors that may play a role in gender differences not only involve genetics and the fact that the X-chromosome contains a higher number of genes involved in host immunity [224], but also differences due to hormone levels and the profile of gut microorganisms [225]. In particular, the hormonal changes occurring during the menstrual cycle seem to affect the response to dietary intervention in a different way [226], while puberty and menopause are life stages characterized by substantial hormonal changes which seem to be related to gut microbes [227,228]. Indeed, one of the vital functions of the gut microbiota is the regulation of steroid hormone levels after menopause, in particular estrogen levels [229], by β-glucuronidase and β-glucosidase enzymes [230]. In addition, gut microbes play a role in host adiposity, as observed in germ-free mice lacking the sexual dimorphism in body fat deposition observed in conventional rodents [231]. In this respect, the study by Yurkovetskiy et al. revealed that the gut microbiota of female adult mice is more similar to the prepubertal gut microbiota composition of both sexes than to male adult mice, suggesting that sex hormones rather than X-chromosome factors were more important in terms of changes to the gut microbiota composition [225]. In addition, type 1 diabetes incidence was similar in germ-free female and germ-free male mice, suggesting that the gut microbiota is a causal factor and not simply a consequence of diabetes [222]. In summary, both hormones and the gut microbiota seem to contribute together in an additive way to the effector mechanisms of type 1 diabetes development [225]. In humans, studies in post-menopausal women have shown higher levels of Firmicutes and lower levels of Actinobacteria, *Lachnospira, Roseburia*, and *Prevotella* compared to premenopausal women, and some bacteria have shown a negative connection between the levels of sexual hormones such as *Prevotella* and estradiol [232]. In humans, the study by Haro et al. showed no differences in α- and β-diversity between sexes, although men showed higher *Veillonella* and *Methanobrevibacter* levels and lower *Bilophila* levels compared to women [233], in agreement with Borgo’s study, which also showed higher levels of *Veillonella* in men, as well as higher levels of Actinobacteria in women [234]. In addition, the levels of *Bacteroides-Prevotella group* were higher in men compared to women in a cross-sectional study [235]. These results were also in agreement with the study by Santos-Marcos et al., which additionally found lower levels of *Sutterella* in women compared to men [232]. The high variability found among studies, possible due to differences in the participants’ ages, the study design and the employed methodology, make it necessary to perform interventional studies separately in men and women in a more personalized way.

#### 5.2.3. Age

The exposure to environmental factors in early stages of life plays a key role in the acquisition of the gut microbiota and impacts the structures of the adult microbial communities and the development of immune-related diseases [8]. The intestinal microbiota is relatively dynamic in the first years of life, with changes depending on the quality of early lifestyle factors. Gut colonization starts before birth in utero with the maternal microbiota, which in turn depends on maternal diet, vaginal health, and antibiotic or drug exposure [236]. Immediately after birth, the colonization process depends on the type of delivery, which will determine bacterial diversity. Particularly, babies born by vaginal delivery acquire a gut microbiota profile more similar to that found in the mother’s vagina, enriched in *Lactobacillus* and *Prevotella* species, whereas cesarean babies are colonized by microbiota from the skin and surroundings, composed of *Staphylococcus* and *Propionibacterium,* among others. The type of feeding also has a great influence on gut communities. Indeed, the gut microbiota from breastfed babies is characterized by higher bacterial diversity compared to formula fed babies [237]. Later on, the gut microbiota becomes more stable and similar to an adult profile around the age of 2.5 years [238] and remains stable during adulthood until elderly age [239]. Indeed, the gut microbiota of healthy adults is characterized by high stability, maintaining a dynamic equilibrium in a constant flux, gaining and losing species over time and with different species having different stabilities. The assembly of some microbes and not others seem to be related to the ability of those specific communities to assemble in the intestine to fill a suite of habitual functional niches providing key metabolic, signalling, and immunomodulatory roles, which may be more stable [5]. Therefore, gut communities remain stable until reaching elderly age, a life stage characterized by a decrease in bacterial diversity and higher levels of proinflammatory bacteria, such as *Fusobacterium, Streptococcus, Staphylococcus* and *Enterobacteria*, and lower levels of immunoregulatory bacteria, such as butyrate producers. Gut microbiota modifications at this stage of life are linked to age-related physiological changes, such as the loss of immune functionality, decreased gut motility, teeth loss, and altered threshold for taste and smell, factors that are related to the reduced intake of fiber-enriched foods, and consequently related to bacteria with the enzymatic ability to fermented carbohydrates [240].

### 5.3. Methodological Factors

There are several factors related to the study design that can influence the quality of the data obtained, as follows: (1) The sample size is a major determinant of reporting reliable statistical results. The bigger the sample size, the more statistical power the study has [241]. (2) Different studies, such as observational or interventional, provide different kinds of data with differing significance in terms of data interpretation. Indeed, observational studies allow data collection, providing an overview of single or multiple points without interfering with the subjects and variables. On the other hand, an interventional study involves a controlled intervention during a period of time in an experimental versus a control group in order to test the effects of a particular bioactive compound in a more controlled setting. Thus, findings from appropriate and well-designed interventional studies are of a causal nature and with stronger scientific evidence than observational ones. (3) The selection of an appropriate control group with the same characteristics as the interventional group and a good placebo product as control. For instance, a common practice in probiotic studies is the use of the same excipient or vehicle as the probiotic capsules, such as maltodextrin for placebo capsules and classic yogurt in the case of probiotic fermented milks studies. The lack of an appropriate control group worsens the quality of the obtained data, since it helps to minimize the effects of all variables, except the interventional product. (4) The methodology used to assess the 16s RNA gene, ranging from traditional molecular approaches to next-generation sequencing technologies, providing a deeper analysis of gut microbial communities [242]. (5) The background diet, and in particular the different composition of the mentioned nutrients or ingredients with immunomodulatory properties. For instance, fiber and probiotic intake constitutes a relevant confounder factor that needs to be controlled in interventional studies in order to avoid biased interpretations. (6) The body composition, and specifically body fat, must be monitored during the study in order to avoid a biased gut microbiota response due to the metabolic consequences associated with body fat changes [243].

### 5.4. Dietary Factors

Besides the dose and duration of the treatment, which represent the major aspects influencing the study results and which are common to all treatments, there are some other factors that depend on the food ingredients or nutrients to be evaluated. We consider the most important ones as follows: (a) Probiotics and yogurts: the type of probiotic strain used, since specificity is a well-known feature of probiotics when referring to immune and intestinal health effects [92], and the vehicle (capsules or fermented products) represent key factors influencing the response of bacterial communities to probiotic intake. In addition, the bacteria content (type of strain for probiotic studies), the FA profile, and bioactive peptides in the case of probiotic fermented milks can differentially impact gut communities. (b) Fiber and polyphenols: the chemical structure, highlighting the polymerization degree, the type of linkage (β or α), the solubility of fibers (soluble or insoluble), and the food matrix used to deliver polyphenols can influence their availability, thus affecting the interaction between polyphenols and coexisting food components during storage and after ingestion [73]. (c) Alcoholic beverages: ethanol, polyphenols, and fiber contents. (d) Sweeteners: the different chemical structures in natural and artificial sweeteners determine their intestinal absorption, and thus their concentration in the gut. (e) Fats: the quantity and quality of the FA profile (SFAs, MUFAs, PUFAs).

## 6. Conclusions

This review aimed to evaluate the extent to which several lifestyle determinants could be involved in the composition of the gut microbiota. The most well-known factors in this regard are diet and stress, and to a lesser extent physical activity, drug intake, and smoking habits. Regarding diet, there are several nutrients and bioactive compounds that can affect the gut microbiota and have been frequently assayed, such as probiotics (including yogurts) and prebiotics (fibers and polyphenols), along with other less-studied but frequently consumed products, such as alcoholic beverages, sweeteners, and fats. The microbiome has becoming a fundamental field to include in the assessment of nutritional status, and hence in the study of human health, especially in particular stages of life or special health conditions. In addition, the balance of gut microbes seems to be pivotal in achieving health preservation in the long term due to its role in the homeostatic processes of the human body. Thus, the study of healthy adults has been promoted as a reference population. However, the high variability and plasticity of the commensal microbiota of healthy adults towards environmental influences and the profound effects of inter- and intra-subject factors complicate the definition of a healthy gut microbiota and the determination of which functions are specifically needed for health preservation, emphasizing the need to stratify the population according to these criteria before the dietary intervention begins. Moreover, the differences in methodological factors found in different studies and the way dietary factors are evaluated need to be homogenized. Furthermore, the great impact of stress on the composition of the gut microbiota through the brain–gut microbiota axis can also influence the initial response to diet, as well as food choices and particular dietary habits, which in turn will also influence the gut microbiota. Future directions involve studying yogurt as a complete immunomodulatory food instead of looking at its individual components; the inclusion of candidate prebiotic fibers such as AXOS and XOS as potential prebiotics; broadening the research into Lactobacilli changes in probiotic and prebiotic studies and the promising short- and medium-chain saturated fatty acids; and continuing the exploration of the effects of ingredients consumed in large quantities in the habitual diet that are under-researched in healthy adults, such as non-alcoholic beer, polyols, and natural and artificial sweeteners. Furthermore, the inclusion of drug consumption, place of living, level of physical activity, and smoking habits in large interventional studies should be included in order to move toward a deeper understanding of the precise lifestyle recommendations needed to maintain gut and immune health and prevent disease development.

## Figures and Tables

**Table 1:** Evidence from human studies relative to yogurt and probiotics effects on the gut microbiota composition of healthy adults.

Ref.	Treatment	Study Type	Study Subjects	Analytic Technique	Results	Results
	Yogurt/Probiotic vs. Control (C)				Differences vs. Basal Point	Differences vs. Control (C)
**Yogurt**						
[37]	Yogurt (10^8^ cfu/g)	Randomized, parallel	15 (6 W/9 M)	Culture + PCR	↔*Lactobacillus* strains	--
					↑*Clostridium coccoides*–*Eubacterium rectale* group	
	Duration: 20 d			Sequence-specific SSU rRNA cleavage with oligonucleotides	↓*Bacteroides*–*Prevotella* group	
	*C: Comparison to basal point*		Age: 24–46 y		↔*Bifidobacterium*, *C. leptum*, *Atopobium*, *Eggerthella*, *Collinsella*, *Lactobacillus*, *Enterococcus*	
			BMI: No data			
[38]	Yogurt (10^7^–10^8^ cfu/g)	Randomized, DB, crossover	79 (47 W/32 M)	DGGE	↑*Lactobacillus*	↔No significant changes
				qPCR	↓*Bacteroides-Porphyromonas-Prevotella*	
	Duration: 4 wks		Age: 24 y			
	*C: Pasteurized yogurt*		BMI: No data			

**Capsules**						
[39]	*Lactobacillus salivarius* CECT5713 (2 × 10^8^ cfu/d)	Randomized, DB,	40 (20 W/20 M)	Culture	↑*Lactobacillus*	↑*Lactobacillus*
	Duration: 6 wks	placebo-controlled, parallel	Age: 33 y			
	*C: Maltodextrin*		BMI: No data			
[40]	(1) *Lactobacillus paracasei* ssp. *paracasei* CRL-431 (10^11^ cfu/d)	Randomized, DB,	71 (46 W/25 M)	Culture	↔*Bacteroides*, *Bifidobacterium*, Clostridia, *Enterobacteriaceae*, *Enterococcus*, *Lactobacillus*	↔*Bacteroides*, *Bifidobacterium*, Clostridia, *Enterobacteriaceae*, *Enterococcus*, *Lactobacillus*
		placebo-controlled, parallel				
	(2) *Bifidobacterium animalis* subsp. *lactis* BB-12 (10^11^ cfu/d)		Age: 26 y			
			BMI: No data			
	Duration: 3 wks					
	*C: Dextrose*					
[41]	*Lactobacillus Zhang* (10^10^ cfu/d)	Randomized, parallel	24 (13 W/11 M)	16s RNA gene Pyrosequencing	↑β-diversity (Unifrac), *Bifidobacterium, Fecalibacterium, Prevotella*	--
	Duration: 4 wks		Age: 41 y	(V5–V6 regions,	↓*Blautia coccoides, Phascolarctobacterium,*	
			BMI: 19.5–28.2 kg/m^2^			
	*C: Comparison to basal point*			Roche)	*Enterobacter*	
				qPCR		
[42]	*Lactobacillus paracasei* DG (2.4 × 10^13^ cfu/d)	Randomized, DB,	34 (19 W/15 M)	16s RNA gene Sequencing	↔α-diversity (Chao1, Shannon)	↑*Coprococcus*
	Duration: 4 wks	placebo-controlled, crossover			↑β-diversity (Unifrac)	↓*Proteobacterias, B. coccoides*
	*C: Maltodextrin + starch*		Age: 35 y	(V3 region, Ion Torrent)		
			BMI: 20–25 kg/m^2^			
[43]	*Lactobacilluscasei* (10^6^–10^8^ cfu/d); *Lactobacillu* *brevis* (10^6^–10^8^ cfu/d); *Bifidobacterium longum +* *Lactobacillus* *lactis + Streptococcus thermophilus* (10^6^–10^8^ cfu/d); *Lactobacillus* *rhamnosus* (10^6^–10^8^ cfu/d); *Lactobacillus* *delbrueckii + St. thermophilus* (10^6^–10^8^ cfu/d); *B. animalis +* *Lactobacillus* *delbrueckii + St. thermophilus* (10^6^–10^8^ cfu/d). Duration: 8 wks	Randomized, parallel	18 (12 W/6 M)	16s RNA gene Pyrosequencing	↔β-diversity (Unifrac)	--
					↑Firmicutes species after *Lactobacillus* and *Bifidobacterium* intake, Bacteroidetes species after *Bifidobacterium* intake	
			Age: 22 y	(V1-V2 regions, Roche)		
			BMI: No data			
	*C: Comparison to basal point*					
[44]	*Bifidobacterium longum* BB536 (4 × 10^8^ cfu/d) + *L. rhamnosus* HN001 (10^9^ cfu/d)	Randomized	16 (4 W/12 M)	qPCR	↔α-diversity (Chao1, Shannon, Simpson)	--
				16s RNA gene Sequencing	↓Firmicutes*, *Proteobacterias	
			Age: 36 y		↑*Blautia* *producta*, *Blautia wexlerae*, *Haemophilus ducrey*	
				(V2-V4-V8, V3-V6, V7-V9 regions, Ion Torrent)	↓*Holdemania filiformis*, *Eubacterium. vulneris*, *Gemminer formicilis*, *Streptococcus sinensis*	
	Duration: 1 month					
			BMI: 20–26 kg/m^2^			
	*C: Comparison to basal point*					
[45]	*Lactobacilluskefiri LKF01* (10^10^ cfu/d)	Randomized	20 (16 W/4 M)	qPCR	↔α-diversity (Chao1, Shannon, Simpson)	--
	Duration: 1 month			16s RNA gene Sequencing	↓Firmicutes, Bacteroides, Proteobacteria, *Bilophila* spp, *Butyricicomonas* spp, *Flavonifractor* spp, *Oscillibacter* spp, * Prevotella* spp	
	*C: Comparison to basal point*		Age: 39 y			
				(V2-V4-V8, V3-V6, V7-V9 regions, Ion Torrent)		
			BMI: 18.5–25 kg/m^2^		↑*Lactobacillus*	
[46]	*Bifidobacterium bifidum* strain Bb (3.8 × 10^9^ cfu/d)	Randomized, DB,	27 (13 W/14 M)	16s RNA gene Sequencing	↔α-diversity (Chao1, Shannon, Simpson), β-diversity (Unifrac)	--
			Age: 31 y			
			BMI: No data			
				(V3 region, Ion Torrent)		
		placebo-controlled, crossover			↓*Prevotellaceae*	
	Duration: 4 wks				↑*Rikenellaceae*, *Ruminococcaceae*	
	*C: Maltodextrin*					
[47]	*L. rhamnosus* IMC501 + *L. paracasei* IMC502 (10^9^ cfu/serving)	Randomized, DB,	50 (27 W/23 M)	Cultures	↔ *Clostridium*, *Enterobacteriaceae*, *Bacteroides*	↑*Lactobacillus*, *Bifidobacterium*
		placebo-controlled, parallel		qPCR	↑*Lactobacillus*, *ifidobacterium*	
	Durantion:12 wks		Age: 23–65 y			
	*C: food products without probiotics*		BMI: No data			
**Probiotics Fermented Milks**						
[48]	(1) *Lactobacillus coryniformis* CECT5711 (2 × 10^8^ cfu/d)	Randomized, DB,	30 (15 W/15 M)	Culture	↑*Lactobacillus*	↑*Lactobacillus*
	(2) *L. gasseri* CECT5714 (2 × 10^8^ cfu/d)	placebo-controlled, parallel		RADP		
			Age: 23–43 y			
	Duration: 2 wks		BMI: No data			
	*C: Yogurt*					
[49]	*L. paracasei* ssp. *paracasei* LC01 (2 × 10^8^ cfu/mL)	Randomized, DB,	52 (31 W/21 M)	qPCR	↑*Lactobacillus*, *Roseburia intestinalis*, *Bifidobacterium*, *E. coli*	↑*Lactobacillus*, *R. intestinalis*
		placebo-controlled, parallel				↓*Escherichia coli*
	Duration: 4 wks		Age: 24 y			
	*C: Semi-skimmed milk*		BMI: 19–29 kg/m^2^			
[50]	*L. acidophilus* LA-5 (10^9^ cfu/d) +	Randomized, DB,	58 (38 W/20 M)	qPCR	↑*Bifidobacterium*	↑*Bifidobacterium*
	*B. animalis* ssp.*lactis* BB-12 (10^9^–10^10^ cfu/d)	placebo-controlled, parallel				↑*Lactobacillus*
	Duration: 4 wks		Age: 32 y	Culture	↑*Lactobacillus*	↓*Enterococcus*
			BMI: 20–28kg/m^2^		↓*Enterococcus*	
	*C: Pasterized Yogurt*					
[51]	*L. acidophilus* LA-5 (10^9^ cfu/d) +	Randomized,	58 (38 W/20 M)	T-RFLP	↔β-diversity (Bray-Curtis)	↔β-diversity (Bray-Curtis)
	*B. animalis* ssp.*lactis* BB-12 (10^9^–10^10^ cfu/d)	placebo-controlled, parallel		qPCR	↔*Bacteroides-Prevotella*, *B. coccoides,*	↔*Bacteroides-Prevotella*,
	Duration: 4 wks		Age and BMI: No		*C. leptum*, *Enterobacteriaceae*, *Enterococcus*, *Bifidobacterium*	*B. coccoides*, *C. leptum Enterobacteriaceae,*
						*Enterococcus*, *Bifidobacterium*
	
			data			
	*C: Yogurt*					
[52]	*L. rhamnosus* GG (2.83 × 10^6^ cfu/g)	Randomized, crossover	13 (0 W/13 M)	16s RNA gene sequencing (V3-V4 regions, Illumina)	↔ α-diversity (Shannon, Simpson)	↑*Intestinibacter bartlettii*
	Duration: 2 wks				↓ *Bilophila wadsworthia*	*↓B. kashiwanohense*,
			Age: 24 y			*B. pseudocatenulatum*, *Megasphaera*
	*C: Milk acidified with 2% of D- (+)-glucono-δ-lactone (400 g)*		BMI: 18.5–25 kg/m^2^			
[53]	*B. animalis* ssp*. lactis* BB-12 (10^7^ cfu/mL)	Parallel	150 (No gender data)	16s RNA gene Sequencing	↔α-diversity (Chao1, Shannon)	--
	Duration: 1 month					
	*C: Comparison to basal point*				↑β-diversity (Bray-Curtis), *Bifidobacterium*, *Slackia*, *Streptococcus*, *Catenibacterium*, *Collinsella*, *Adlercreutzia*	
			Age: 18–40 y	(V4 region, Illumina)		
			BMI: 18–28 kg/m^2^			
					↓*Lachnoclostridium, Roseburia, Acidaminococcaceae*	
[26]	*B. animalis ssp.lactis, L. delbrueckii* ssp. *bulgaricus, Lactococcus lactis ssp. cremoris, St. thermophilus* [2 units/d (3.2 × 10^7^ GE + 6.3 × 10^7^ GE)]	Randomized, parallel	14 (14 W/0 M)	16s RNA gene Pyrosequencing	↔No significant changes	--
			Age: 21–32 y	(V2 region, 454 FLX)		
			BMI: No data			
	Duration: 7 wks					
	*C: Comparison to basal point*					
[54]	*L. casei* DN-114001 (10^8^ cfu/mL)	Randomized	12 (7 W/5 M)	qPCR	↑*L. casei*	--
	Duration: 10 d			FISH	↔*C.coccoides, F. prausnitzii, Bacteroides, Bifidobacterium, Atopobium,*	
			Age: 23–44 y			
	*C: Comparison to basal point*		BMI: No data		*Lactobacilli*–*Enterococci*, *Enterobacteria*	

No changes; Not applicable; BMI: Body mass index; cfu: colony forming units; d: day; DB: double-blind; GE: Genomic units; M: Men; qPCR: Quantitative polimerase chain reaction; RADP: Random Amplification of Polymorphic DNA; T-RFLP: Terminal restriction fragment length polymorphism; W: Women; wks: weeks; y: years. ↑: increase ↓: decrease.

**Table 2:** (**a**) Evidence from human studies relative to fiber effects on the gut microbiota composition of healthy adults. (**b**) Evidence from human studies relative to polyphenols effects on the gut microbiota composition of healthy adults.

a. Evidence from human studies relative to fiber effects on the gut microbiota composition of healthy adults							
Ref.	Treatments	Study Type	Study Subjects	Analytic Technique	Results	Results	Results
	Fiber vs. Control (C)				Differences vs. Basal Point	Differences between Treatment Groups	Differences vs. Control (C)
**Accepted Prebiotic Fibers**							
[55]	Agave fructan (5 g/d)	Randomized, DB, placebo-controlled crossover	38 (19 W/19 M)	Colon culture model	↑*Bifidobacterium*, *Lactobacillus-Enterococcus* group	--	↑*Bifidobacterium*, *Lactobacillus-Enterococcus* group
			Age: 35 y				
				FISH			
	DP: 3–30		BMI: 21.1–27.1 kg/m^2^	PCR			
	Duration: 3 wks						
	*C: Maltodextrin*						
[56]	Agave inulin (5 or 7.5 g/d)	Randomized, DB, placebo-controlled crossover	29 (No gender data)	16s RNA gene Sequencing	--	↔No significant changes	(1) 5, 7.5 g: ↑Actinobacterias, *Bifidobacteriaceae*, *Bifidobacterium*,
	DP: 25–34		Age: 27 y				
	Duration: 3 wks		BMI: 18.5–29.5 kg/m^2^	(V4 region, Illumina)			*B. adolescentis*, *B. breve*, *B. longum,*
	*C: Agave inulin (0 g/d)*						*B. pseudolongum*
							↓*Desulfovibrio*
							(2) 7.5 g: ↓*Lachnobacterium*, *Ruminococcus*
[57]	β2–1 fructan	Randomized, DB, placebo-controlled crossover	30 (17 W/13 M)	qPCR	↑*Bifidobacterium*	--	↑*Bifidobacterium*
	(inulin and short-chain oligosaccharides) (15 g/d)		Age: 28.1 y				
			BMI: 21.2–27.2 kg/m^2^				
	Duration: 4 wks						
	*C: Maltodextrin*						
[58]	FOS	Randomized, DB, crossover	(1)FOS: 34	16s RNA gene sequencing	(1) FOS: ↑*Bifidobacterium*	No statistical analysis performed	--
	GOS		(24 W/10 M)		↓*Phascolarctobacterium, Enterobacter, Turicibacter, Coprococcus, Salmonella*		
	(16 g/d)		Age: 21.9 y				
	Duration: 2 wks		BMI: 19.8–26.4 kg/m^2^	(V2 region, Ion Torrent)	↔α-diversity (Shannon)		
	*C: Comparison to basal point*		(2)GOS: 35		(2) GOS: ↑*Bifidobacterium*		
			(25 W/10 M)		↓α-diversity (Chao 1, Shannon, phylogenetic tree), *Ruminococcus*, *Dehalobacterium*, *Synergistes*, *Holdemania*		
			Age: 22.1 y				
			BMI: 19.8–26.4 kg/m^2^				
[59]	HMO (2-O-fucosyllactose (2′FL), lacto-N-neotetraose (LNnT), 2′FL + LNnT)	Randomized, DB, placebo-controlled parallel	100 (49 W/51 M)	16s RNA gene Sequencing (V3-V4 regions, Illumina)	(1) 2′FL (5, 10 g/d), LNnT and 2′FL + LNnT (5, 10, 20 g/d): ↑Actinobacterias	20 g/d: ↑Actinobacterias	(1) 5, 10, 20 g/d: ↑Actinobacterias
			Age: 30–40 y				(2) 2′FL (10 g/d), LNnT (5, 10, 20 g/d), 2′FL + LNnT (10, 20 g/d):
			BMI: 20–28 kg/m^2^				
							↑*Bifidobacterium, B. adolescentis*
	(5, 10 or 20 g/d)				(2) 2′FL (10 g/d): ↓Proteobacterias		(3) 2′FL + LNnT (20 g/d): ↑*B. longum*
	Duration: 2 wks				(3) LNnT and 2′FL + LNnT (20 g/d): ↓Firmicutes		
	*C: Glucose* (2 g)						
[60]	Inulin-type fructan (16 g/d)	Randomized, DB, placebo-controlled crossover	34 (13 W/21 M)	16s RNA gene Sequencing (V3-V4 regions, Illumina)	HDF, LCF: ↑Actinobacterias, *Bifidobacterium*, *Oscillospira*	LDF vs. HDF: ↑*Lactobacillus*	↑Actinobacterias, *Bifidobacterium*, *Oscillospira*
	Duration: 3 wks		Age: 37 y				
	*C: Maltodextrin (16 g/d)*		BMI: 20–−27 kg/m^2^		↔α and β-diversity	HDF vs. LDF: ↑*Ruminococcaceae*, *Fecalibacterium*	↓Firmicutes, *Dorea*, *Coprococcus*, *Ruminococcus*
			High dietary fiber (HDF) or Low dietary fiber (LDF)		HDF: ↑*Bifidobacterium*, *Fecalibacterium*		
							↔α-diversity (Shannon, Chao1),
					↓Firmicutes, *Dorea*, *Coprococcus*, *Ruminococcus*		β-diversity (Unifrac)
					LCF: ↑*Bifidobacterium*		
[61]	Very long chain inulin (10 g/d)	Randomized, DB, placebo-controlled crossover	32 (18 W/14 M)	FISH	↑*Bifidobacterium*	--	↑*Bifidobacterium*, *Atopobium*
			Age: 25 y		*Lactobacillus-Enterococcus*		*Lactobacillus-Enterococcus*
	Duration: 3 wks						↓*Bacteroides-Prevotella*
	*C: Maltodextrin (10 g/d)*		BMI: 20–30 kg/m^2^				↔*E.coli*, *E. rectale-C. coccoides*, *Ruminococcus*
**Candidate Prebiotic Fibers**							
[62]	RMD (15 or 25 g/d)	Randomized, DB, placebo-controlled crossover	49 (28 W/21 M)	qPCR	↔ Bifidobacteria and total bacteria	No statistical analysis performed	(1) 15 g/d: ↔No significant changes
	Duration: 3 wks		Age: 26 y				
			BMI: 21–28 kg/m^2^				(2) 25 g/d: ↑Bifidobacterias
	*C: Maltodextrin*						
[63]	RPS (30 g/d)	Randomized, DB, placebo-controlled	42 (24 W/18 M)	qPCR	↑*R. bromii*, *Bifidobacterium,*	--	↑*Bifidobacterium*
	Duration: 12 wks		Age: 42 y	16s RNA gene Sequencing			↓α-diversity
					*B. ruminantium*		(Shannon, Inverse Simpson)
	*C: Corn starch*		BMI: No data	(V4 region, Illumina)	↓*R. obeum*, *R. torques*, *B. dentium*		
[64]	XOS (8 g/d)	Randomized, DB, placebo-controlled crossover	41 (20 W/21 M)	FISH	--	--	↑*Bifidobacterium*
	Duration: 3 wks		Age: 43 y	Flow cytometry			↔*Bacteroides/Prevotella*, *Clostridium* I and II, *Lactobacillus/Enterococcus Atopobium*, *B. lactis*
	*C: Maltodextrin*		BMI: 20–30 kg/m^2^				
[65]	AXOS-enriched Bread (2.2 g/d)	Randomized, DB, placebo-controlled crossover	40 (20 W/20 M)	FISH	↑*Bifidobacterium*, *Bacteroides*	--	--
	Duration: 21 d		Age: 31 y				
					*Lactobacillus*		
	*C: Non-endoxylanase treated breads*		BMI: 20.−26 kg/m^2^				
[66]	Polydextrose (PDX)	Randomized, DB, placebo-controlled crossover	21 (0 W/21 M)	16s RNA gene Pyrosequencing (V3-V4 regions, Roche)	--	↔α-diversity (Shannon, Chao1)	SCF, PDX: ↑*Clostridiaceae*, *Veillonellaceae*, *Fecalibacterium,* ↓Actinobacteria and Firmicutes
	Soluble Corn Fiber (SCF) (21 g/d)		Age: 27 y			SCF vs. PDX: ↑Proteobacterias. *Lactobacilli*, *Alcaligenaceae*, *Roseburia*,↓*Oscillospira,*	
	Duration: 21 d		BMI: 23–31 kg/m^2^				
							↔α-diversity (Shannon, Chao1)
	*C: No supplemental fiber (NFC)*					PDX vs. SCF: ↑Verrucomicrobia, *Clostridium*, *Akkermansia,*	
							SCF: ↑Proteobacteria, *Lactobacilli*
							PDX: ↑*Clostridium*, *Akkermansia,*
						↓*Lachnospiraceae*	
							*C. leptum*, ↓*Hyphomicrobiaceae*
**Mixed Accepted and Candidates Prebiotic Fibers**							
[67]	(1) RPS (28–34 g/d)	Randomized, DB, placebo-controlled, parallel	174	qPCR	(1) RPS: ↑*B. fecale/adolescentis/stercoris*	No statistical analysis performed	--
					(2) RMS: ↑*R. bromii*		
				16s RNA gene Sequencing			
					(3) Inulin: ↑*Anaerostipes hadrus*,		
			Age: 19 y		*B. fecale/adolescentis,*		
					*B. longum/breve,*		
	(2) RMS (20–24 g/d)		Gender and BMI: no data	(V4 region, Illumina)	*B.catenulatum/pseudocatenulatum/*		
	(3) Inulin (20 g/d)				*kashiwanohense*, *B. bifidum*, *E. rectale*		
	Duration: 2 wks						
	*C: Amylase-accessible corn starch*						
[68]	(1) XOS (5 g/d)	Randomized, DB, placebo-controlled, parallel	65 (33 W/26 M)	qPCR	XOS + inulin: ↑*Lactobacillus*	No statistical analysis performed	XOS + inulin/XOS: ↑*Bifidobacterium* (V2, V3), *Peptostreptococcus* (V2)
	(2) XOS (1 g/d) + Inulin				↔Firmicutes spp, Bacteroidetes spp, *Clostridium*, *Staphylococcus*, *Eubacterium*, *Peptostreptococcus*, *Fusobacterium*, *Enterobacterium*, *F. prausnitzii*, *Roseburia* spp.		
			Age: 18–24 y				
	(chicory) (3 g/d)		BMI: 18.5–27 kg/m^2^				
	DP inulin: 10						
	Duration: 4 wks						↔Firmicutes spp, Bacteroidetes spp, *Clostridium, Staphylococcus, Eubacterium, Fusobacterium, Enterobacterium,*
	*C: Maltodextrin*						
							*F. prausnitzii*, *Roseburia* spp.
**Dietetic Fibers**							
[21]	Dietetic fiber (10 or 40 g/d)	Randomized, crossover	19 (10 W/9 M)	qPCR	(1) 40 g/d: ↓*E. coli*	No statistical analysis performed	40 g/d: ↑Microbial change (JSD metrics) in subjects with a low richness
				16s RNA gene Pyrosequencing (V3-V4 regions, Roche)	(2) 10, 40 g/d: ↔*C. coccoides*, *C. leptum*, *Bacteroides-Prevotella*, *Bifidobacterium*		
	Duration: 5 d		Age: 19–25 y				
	*C: Comparison to basal point*		BMI: 18.5–25 kg/m^2^				
[69]	(1) High Whole Grain (WG) Diet (>80 g/d WGs)	Randomized, crossover	33 (21 W/12 M)	FISH	↔No significant changes	↔No significant changes	--
			Low consumers of WG diet				
	(2) Refined grain diet (<16 g/d WGs)						
			Age: 49 y				
	Duration: 6 wks		BMI: 20–35 kg/m^2^				
	*C: Comparison to basal point*						
[70]	Whole Grain Diet	Randomized, controlled, parallel	81 (32 W/49 M)	16s RNA gene Sequencing	↔No significant changes	--	↑*Lachnospira*
	(16 g fiber/1000 kcal)		Age: 54–55 y	(V4 region, Illumina)			↓*Enterobacteriaceae*
	Duration: 6 wks		BMI: 20–35 kg/m^2^				↔α-diversity (phylogenetic tree),
	*C: Refined grain diet*						β-diversity (Unifrac)
	*(8 g fiber/1000 kcal)*						
**b**. Evidence from human studies relative to polyphenols effects on the gut microbiota composition of healthy adults							
**Ref.**	**Treatments**	**Study Type**	**Study Subjects**	**Analytic Technique**	**Results**	**Results**	**Results**
	**Polyphenols vs. Control (C)**				**Differences vs. Basal Point**	**Differences between Treatment Groups**	**Differences vs. Control (C)**
[71,72]	Wild blueberry drink	Randomized, DB, placebo-controlled crossover	15 (0 W/15 M)	qPCR	↑*Bifidobacterium*	--	--
					↔*Bacteroides*, *Prevotella*,		
					*Enterococcus*, *C. coccoides,*		
					*Bifidobacterium* species		
					↑*B. longum* subsp. *infantis*		
	(25 g/250 mL)		Age: 47 y				
			BMI: 22–28 kg/m^2^				
	[Chlorogenic acid (127.5 mg) + anthocyanins (375 mg)]						
	Duration: 6 wks						
	*C: Placebo drink*						
[73]	Boysenberry juice	Randomized, placebo-controlled crossover	24 (5 M/20 W)	qPCR	--	↔*Bacteroides-Prevotella-*	↔*Bacteroides-Prevotella*-
	(anthocyanins, ellagitannins and ellagic acid derivatives; 750 mg)		Age: 50 y				*Porphyromonas* group,
						*Porphyromonas* group,	*Bifidobacterium*,
							*C. perfringens, Lactobacillus*
			BMI: 18–35 kg/m^2^			*Bifidobacterium*,	
						*C. perfringens*, *Lactobacillus*	
	Duration: 4 wks						
	*C: Placebo drink*						
[74]	Fruits and Vegetables (2 (6 wks), 4 (12 wk) and 6 portions (18 wks))	Randomized, controlled, parallel	122 (74 W/48 M)	FISH	(1) HF: ↑B*acteroides/Prevotella*	No statistical analysis performed	LF: ↑*Bifidobacterium*
			Age: 49–52 y		(2) LF: ↑*Bifidobacterium*,		
			BMI: 18–35 kg/m^2^		B*acteroides/Prevotella,*		
	Duration: 18 wks		High-flavonoid (HF)/Low-flavonoid (LF)		*C. leptum-R. bromii/flavefaciens*		
	*C: Habitual diet*						
[75]	Cocoa flavanols	Randomized, DB, crossover	22 (12 M/10 W)	FISH	(1) HCF: ↑*Bifidobacterium, Lactobacillus*, *Enterococcus*	--	HCF: ↑*Bifidobacterium, Lactobacillus*, *Enterococcus*
	(catechin, epicatechin, theobromine) HCF: High–cocoa flavanol group; 494 mg/d						
			Age: 30 y				
	Duration: 4 wks		BMI: 20–25 kg/m^2^		↓*C. histolyticum*		
	*C: Low–cocoa flavanol (LCF) group (23 mg/d)*				(2) LCF: ↑*E. rectale-C. coccoides group, C. histolyticum*		
							↓*C. histolyticum*
[76]	Green tea [400 mL/d (100.2 μg gallic acid Eq/mL)]	Intervention	12 (4 W/8 M)	16s RNA gene Sequencing	↑α-diversity (Simpson, Shannon and Chao1), ↑Actinobacteria, Firmicutes,	--	--
			Age: 34 y				
				(V4-V5 regions, Illumina)			
					Butyrate-producing bacteria,		
			BMI: 18–24 kg/m^2^		↓Bacteroidetes members		
	Duration: 2 wks						
	*C: Comparison to basal point*						
[77]	Green Tea	Randomized, single blind, placebo-controlled parallel	58 (46 W/12 M)	16S–23S rDNA Intergenic spacer region	↔α-diversity (Shannon), Actinobacteria, Firmicutes,	--	↔α-diversity (Shannon), Actinobacteria, Firmicutes,
	(>1.35 g Catechins; >0.56 g Epigallocatechin-3-gallate)		Age: 29 y				
							Bacteroidetes, Fusobacteria, Verrucomicrobia, Proteobacteria
					Bacteroidetes, Fusobacteria, Verrucomicrobia, Proteobacteria		
			BMI: 18–25 kg/m^2^				
	(9 capsules/d)						
	Duration: 12 wks						
	*C: Microcrystalline cellulose*						

↔ No changes; Not applicable; 2′FL: 2′-O-fucosyllactose; BMI: body mass index; DB: double-blind; DP: Degree of polymerization; FISH: Fluorescent in situ hybridization; FOS: fructo-oligosaccharides; GOS: Galacto-oligosaccharides; HMO: Human milk oligosaccharides; JSD: Jensen Shannon Distances; LNnT: lacto-N-neotetraose; M: Men; PCR: Polimerase chain reaction; RMD: Resistant maltodextrin; RPS: resistant potato starch; RMS: resistant maize starch; XOS: Xilo-oligosaccharides; W: Women; WG: Whole grain; wks: weeks. y: years. ↑: increase; ↓: decrease.

**Table 3:** Evidence from human studies relative to alcoholic beverages effects on the gut microbiota composition of healthy adults.

Ref.	Treatments	Study Type	Study Subjects	Analytic Technique	Results	Results	Results
	Alcohol vs. Control (C)				Differences vs. Basal Point	Differences between Treatment Groups	Differences vs. Control (C)
[78]	Red wine (100 mL/d)	Observational	38 (27 W/11 M)	qPCR	--	--	↓*Bifidobacterium*,
			Age: 55–67 y				*B. coccoides*, *C. leptum, Lactobacillus*
	*C: Non-wine consumers*		BMI: 22–30 kg/m^2^				
[79]	Vodka (2 mL in 300 mL orange or strawberry juice)	Observational	15 (4 W/11 M)	16s RNA gene Sequencing	↔α-diversity (Chao1), β-diversity (Bray-Curtis)		
					↔ Main phyla, families, genera and species analyzed		
			Age: 26 y	(V1-V2 region, Illumina)			
			BMI: 23–27 kg/m^2^				
	*C: Comparison to basal point*						
[80]	(1) RW (Red wine; 272 mL/d)	Randomized, controlled, crossover	10 (0 W/10 M)	PCR	(1) RW: ↑*Enterococcus, Prevotella, Bacteroides, B. uniformis, Bifidobacterium,*	Gin vs. RW and DRW:	--
			Age: 48 y	DGGE + qPCR			
					*E. lenta*, *B. cocoides-E. rectale group*	↑*Clostridium,*	
					(2) DRW: ↑*Enterococcus*, *Bifidobacterium*, *E. lenta,*	*C. histolyticum*	
						*↓Prevotella*, *Bifidobacterium*, *Enterococcus*, *E. lenta*	
	(2) DRW (Dealcoholized red wine; 272 mL/d)		BMI: 24.6–30.8 kg/m^2^		*B. cocoides-E. rectale group*		
	(3) Gin (100 mL/d)						
	Duration: 20 d						
	*C: Comparison to basal point*						
[81]	Red wine	Randomized, controlled, parallel	20	16s RNA gene Sequencing	↑α-diversity (Shannon-Weaver), *Slackia*, *Gordonibacter*, *Oscillatoria*, *Veillonela*	--	--
	(272 mL/d)		Age: 20–48 y				
	Duration: 1 month		Gender and BMI: No data	(V1-V2 regions, Illumina)			
	*C: Non-wine consumers*						
[82]	(1) AB: Alcoholic beer	Interventional, 2 phases study, parallel	NAB: 35 (14 W/21 M)	16s RNA gene Sequencing	(1) AB: ↑Bacteroidetes, *Dysgonomonas*, *Pseudomonas*, *Succinivibrio*	No statistical analysis performed	--
	(355 mL/d)		Age: 21–53 y		↓Firmicutes		
	(2) NAB: Non-alcoholic beer				(2) NBA: ↑α-diversity (Chao1, Shannon), β-diversity (Unifrac),		
			AB: 33 (15 W/18 M)				
				(V3 region, Roche)	↑Bacteroidetes, *Dialister*, *Actinomyces*, *Staphylococcus*, *Parabacteroides*, *Veillonella*, *Haemophilus*, *Lactococcus*, *Bacteroides*, *Weissella*, *Phascolarctobacterium*, *Streptococcus*, *Acinetobacter*, *Sutterella*, *Turicibacter*, *Lactobacillus*		
	(355 mL/d)		Age: 21–55 y				
	Duration: 30 d						
	*C: Comparison to basal point*		BMI: No data				
					↓Firmicutes		

↔ No changes; Not applicable; AB: Alcoholic beer; BMI: Body mass index; DGGE: Denaturing gradient gel electrophoresis; DRW: Dealcoholized red wine; M: Men; NAB: Non-alcoholic beer; qPCR: Quantitative Polymerase chain reaction; RW: Red wine; W: Women; y: years. ↑: increase; ↓: decrease.

**Table 4:** Evidence from human studies relative to sweeteners effects on the gut microbiota composition of healthy adults.

Ref.	Treatments Sweeteners vs Control (C)	Study type	Study subjects	Analytic technique	Results Differences vs. basal point	Results Differences vs. Control (C)
[83]	Isomalt Dose: 30 g/d Duration: 4 wk *C: Sucrose*	Randomized, DB, placebo-controlled, crossover	19 (12 W/7 M) Age: 35 y BMI: 23-26 kg/m^2^	Culture FISH	--	↑Bifidobacteria ↑*Atopobium*, Actinobacteria ↓*Roseburia intestinalis*, *Bacteroides*
[84]	Lactitol Dose: 0, 5 or 10 g/d Duration: 1 wk *C: Sucrose*	Randomized, DB, placebo-controlled, longitudinal	75 (26 W/39 M) Age: 18-24 y BMI Women: 20-25 kg/m^2^ BMI Men: 20-26 kg/m^2^	Culture	(1) 5 g: ↔No significant changes (2) 10 g: ↑*Bifidobacterium* ↔Bacterial counts of total anaerobes, aerobes, *Enterobacteriaceae*, Lactobacilli	--
[85]	(1) Aspartame (2) Acesulfame-K Data collection: 24 h recalls *C: Non-consumers*	Observational	31 (20 W/11 M) Age: 27 y BMI: 20-28 kg/m^2^	LH-PCR	--	↑β-diversity (Unifrac) ↔No significant changes at class and order level
[86]	Non-caloric artificial sweeteners (NAS) Data collection: FFQ	Observational	172 Age: 43 y Gender and BMI: No data	16sRNA gene Sequencing (V2 region, Illumina)	Positive correlations with Actinobacteria, *Enterobacteriaceae*, Deltaproteobacteria	--
	ccharin Dose: 120 mg/d Duration: 6 d *C: Comparison to basal point*	Intervention	7 (2 W/5 M) Age: 28-36 y BMI: No data	16sRNA gene Sequencing (V2 region, Illumina)	Different bacteria clustering between NAS groups (1) NAS Responders: ↑Lactobacillales, Bacteroidales ↓Clostridiales (2) NAS non-Responders: ↔No significant changes	--

↔ No changes; -- Not applicable; BMI: Body mass index; d: day; DB: double-blind; FFQ: Food Frequency Questionnaire; FISH: Fluorescent in situ hybridization; LH-PCR: Length heterogeneity polymerase chain reaction; M: Men; NAS: Non-caloric artificial sweeteners; W: Women; wk: week; y: years.

**Table 5:** Evidence from human studies relative to fats effects on the gut microbiota composition of healthy adults.

Ref.	Treatments	Study Type	Study Subjects	Analytic Technique	Results	Results	Results
	Fats vs. Control (C)				Differences vs. Basal Point	Differences between Treatment Groups	Differences vs. Control (C)
[87]	Soybean Oil diet	Randomized, parallel	217 (114 W/103 M)	16s RNA gene Sequencing	(1) Low fat: ↑*Blautia, Fecalibacterium*	(1) Low vs. High: ↑α-diversity (Shannon index)	--
	Low (Fat: 20% total energy), Medium (30%) and High (40%)						
			Age: 23 y	(V3-V4 regions, Illumina)	(2) Medium fat: ↑Bacteroidetes	(2) High vs. Low: ↑Bacteroidetes, *Alistipes*, *Bacteroides*	
			BMI: 19–24 kg/m^2^				
					(3) High fat: ↑Bacteroidetes, *Alistipes*, *Bacteroides*		
	Duration: 6 months					↓Firmicutes, *Blautia*, *Fecalibacterium*	
	*C:Comparison to basal point*				↓Firmicutes, *Fecalibacterium*		
[88]	Saturated fat (dairy or butter)	Randomized, controlled, parallel	109 (No gender data)	16s RNA gene Sequencing	--	--	↔α-diversity (Shannon),
	(15% total energy)						β-diversity (Unifrac)
				(V4 region, Illumina)			Changes in 57 bacterial genus
	Duration: 4 wks		Age: 21–65 y				
	*C: Low fat diet (7% saturated fat)*		BMI: 18–36 kg/m^2^				
[89]	Omega-3 Drink (D) or capsules (C) (2000 mg/d DHA + 2000 mg/d EPA)	Randomized, DB, crossover	22 (12 W/10 M)	16s RNA gene Sequencing	D and C: ↔α-diversity (Shannon index), β-diversity (Unifrac)	D: ↑*Lachnospira, Roseburia*	--
			Age: 51–65 y				
			BMI: 22–34 kg/m^2^	(V4 region, Illumina)			
	Duration: 8 wks				↑*Clostridiaceae*, *Sutterellaceae*, *Akkermansiaceae*, *Oscillospira*, *Lachnospira*, *Bifidobacterium*, *Lactobacillus*		
	*C: No control group.*						
	*Comparison to basal point*						
					↓*Coprococcus*, *Fecalibacterium*		
[90]	Dairy Cream (48% SFA) (341 mL/d)	Randomized, parallel	25 (0 W/25 M)	16s RNA gene Sequencing	↔α-diversity (Shannon),	--	--
			Age: 23 y		β-diversity (Unifrac)		
	Duration: 1 wk		BMI: 21–25 kg/m^2^	(V4 region, Illumina)	↑Betaproteobacterias, ↓*Bacteroidaceae*		
	*C: No control group.*						
	*Comparison to basal point*						
[91]	(1) Semi-skimmed ewe’s milk yogurt (ES)(2.8% fat) (250 g/d)	Randomized, DB, crossover	30 (16 W/14 M)	qPCR	--	(1) CW vs. ES: ↑*C. leptum* group	--
			Age: 42 y				
	(2) Whole ewe’s milk yogurt		BMI: 19–28 kg/m^2^			↔*Bacteroides*, *F. prausnitzii,*	
	(EW) (5.8% fat) (250 g/d)					*Bifidobacterium* spp., *Lactobacillus* spp., *Enterobacteriaceae*,	
	Duration: 5 wks						
	*C: Cow whole’s milk yogurt (CW) (3.0% fat*) (250 g/d)					*Enterococcus* spp	
						(2) Women (Highest ratio of total cholesterol/HDL–cho): EW vs. ES:	
						↓*B. coccoides-E. rectale*	

↔ No changes; Not applicable; BMI: Body mass index; CW: Cow’s milk yogurt; DB: double-blind; DHA: Docosahexaenoic acid; EPA: Eicosapentaenoic acid; ES: Semi-skimmed milk yogurt; EW: whole ewe’s milk yogurt; M: Men; SFA: Saturated fatty acids; W: Women; wk: week; y: years. ↑: increase; ↓: decrease.
